# Quantification of phospholipids and glycerides in human milk using ultra-performance liquid chromatography with quadrupole-time-of-flight mass spectrometry

**DOI:** 10.3389/fchem.2022.1101557

**Published:** 2023-01-09

**Authors:** Yan Liu, Weicang Qiao, Yanpin Liu, Junying Zhao, Qian Liu, Kai Yang, Minghui Zhang, Yaling Wang, Yan Liu, Lijun Chen

**Affiliations:** ^1^ National Engineering Research Center of Dairy Health for Maternal and Child, Beijing Sanyuan Foods Co. Ltd., Beijing, China; ^2^ Beijing Engineering Research Center of Dairy, Beijing Technical Innovation Center of Human Milk Research, Beijing Sanyuan Foods Co. Ltd., Beijing, China

**Keywords:** UPLC-Q-ToF-MS, human milk, lipids, phospholipids, glycerides, gangliosides, quantification

## Abstract

Human milk lipids, which are an important source of energy and affect growth and development of infants, require a comprehensive method for its qualitative and quantitative analysis. This work describes a method for the analysis of phospholipids, glycerides, free fatty acids and gangliosides in human milk by ultra-performance liquid chromatography using a C18 column with quadrupole-time-of-flight mass spectrometry (Q-TOF-MS). The lipids were extracted by liquid-liquid extraction and phospholipids were separated by solid phase extraction (SPE). The chromatographic columns with two different specifications (4.6 mm × 150 mm, and 3 mm × 50 mm) were used to detect phospholipids and glycerides in human milk, respectively. The sphingolipids and glycerides were analyzed in positive ion mode, and the glycerophospholipids and free fatty acids were analyzed in negative ion mode. Both internal and external standards were used for absolute quantification in this experiment. 483 species of lipids, including phospholipids, glycerides, free fatty acids and gangliosides, in human milk were analyzed using UPLC-Q-TOF-MS with high sensitivity and good linearity, with coefficient of correlation above 0.99, the relative standard deviation of accuracy and precision less than 10%. The results in a large number of human milk samples showed that this method was suitable for qualitative and quantitative analysis of lipids in human milk, even for other mammalian milk and infant formulae.

## 1 Introduction

Lipids are generally defined as a group of organic compounds in living organisms, most of which are insoluble in water but soluble in non-polar organic solvents. Lipids are one of the important nutrients that supply energy and essential fatty acids for the body. Fahy, et al. (2008) categorized lipids into eight classes: fatty acids, glycerides, glycerophospholipids, sphingolipids, sterol lipids, prenol lipids, saccharolipids and polyketides. For human milk, lipids account for 3%–5%, which are the second largest component including 98%–99% glycerides, 0.26%–0.80% phospholipids (including glycerophospholipids and sphingolipids), 0.08%–0.40% free fatty acids, 0.25%–0.34% cholesterol and 0.001% gangliosides (Delplanque et al., 2015; Zou et al., 2017; George et al., 2018). Studies have shown that the composition of human milk lipids affects growth and development of infants and young children. As for infants and young children, 45%–55% of daily energy is provided by lipids (Caudill, 2010; Contarini and Povolo, 2013). Phospholipids are an indispensable component of cell membrane and organelle membrane, and can resist various metabolic diseases (Verardo et al., 2017). Human milk lipids also play an important role in infants’ cell proliferation, small intestine cell maturation, intestinal function, nerve and brain development, immunity and resistance to pathogen invasion, signal transduction, etc., (Sánchez-Juanes et al., 2009; Gallier et al., 2010; Koletzko, 2016). Therefore, it is necessary to comprehensively profile the lipids in tissue samples.

Presently, the traditional liquid-liquid extraction (LLE) methods, including Folch method (Folch et al., 1957) and Bligh & Dyer method (Bligh and Dyer, 1959), are the commonly used methods for lipids extraction, in which chloroform and methanol are both involved. Dichloromethane is also used in some studies (Castro-Gómez et al., 2014; Claumarchirant et al., 2016; Liaw et al., 2016; Yangbo, 2016). Several classes of lipids were extracted by LLE method separately, and the all classes of lipids including phospholipids, glycerides, free fatty acids and gangliosides were extracted by different human milk sample (McJarrow et al., 2019; Liu et al., 2020; Xu et al., 2020; Song et al., 2021; Zhang et al., 2021). Recently, liquid chromatography-mass spectrometry (LC-MS) has been widely used in lipids analysis. The technology of electronic spray ionization-mass spectrometry (ESI-MS) has been widely used in the analysis of lipids for the structure information (Han and Gross, 1994; Han and Gross, 2001; Schwudke et al., 2006). Time of flight-mass spectrometry (TOF-MS) has the advantages of fast scanning speed, fast ion transfer rate and high sensitivity. With the combination of the above two technologies, the accuracy of profiling lipid in complex samples could be effectively improved (Mirsaleh-Kohan et al., 2008; Zhao et al., 2021). Presently, most of the studies extract and detect glycerides, phospholipids and gangliosides by TOF-MS, respectively (Ten-Domenech et al., 2015; Ali et al., 2017; Tu et al., 2017; Jiang C. et al., 2018; George et al., 2020; Bukowski and Picklo, 2021; Song et al., 2021).

Human milk is the gold standard for design of infant formula. Because human milk lipids are affected by maternal genes, diet, environment and other factors (Liaw et al., 2016; Vieira et al., 2018), it is necessary to establish the database of human milk lipids by profiling their representative samples. However, due to the small collection volume of human milk, especially colostrum, the establishment of a human milk lipid database relies on methods which could extracted phospholipids, glycerides, free fatty acids and ganglosides. Therefore, it is necessary to study low volume of human milk with the methods.

This study aims to separate and detect human milk lipids with a method that combines solid phase extraction (SPE), ultra-performance liquid chromatography/quadrupole-time-of-flight mass spectrometry (UPLC-Q-TOF-MS), and electrospray ionization source (ESI). This method enables qualitative and quantitative analysis of lipids, including free fatty acids (FFA), triacylglycerols (TAG), diacylglycerols (DAG), phosphatidylcholine (PC, including LPC (lysophosphatidylcholine)), phosphatidylethanolamine (PE, including LPE (lysophosphatidylethanolamine)), phosphatidylinositol (PI, including LPI (lysophosphatidylinositol)), phosphatidylserine (PS, including LPS (lysophosphatidylserine)), phosphatidylglycerol (PG, including LPG (lysophosphatidylglycerol)), phosphatidic acid (PA, including LPA (lysophosphatidic acid)), sphingomyelin (SM), ceramides (Cer) and ganglioside (GM3 and GD3). This study is intended to provide the method for the analysis of human milk lipids at molecular level and serve as a reference for the subsequent optimization of infant formulae.

## 2 Materials and methods

### 2.1 Chemicals and reagents

Methanol, acetonitrile, isopropanol, dichloromethane, chloroform and ammonium acetate were all of LC-MS grade, and purchased from Thermo Fisher Scientific (United States). The standards of phospholipids, including phosphatidylcholine (PC, 18:0/18:2 and 14:1/17:0), phosphatidylethanolamine (PE, 18:0/18:2 and 14:1/17:0), phosphatidylinositol (PI, 16:0/18:1 and 14:1/17:0), phosphatidylglycerol (PG, 16:0/18:1 and 14:1/17:0), sphingomyelin (SM, d20:1/24:0 and d17:0/18:1) and ceramide (Cer, d18:1/24:1 and d18:2/24:0), were purchased from Avanti Polar Lipids (United States). The standards of glycerides, including triacylglycerols (TAG, 16:0/18:1/18:1 and d5-18:1/18:1/18:1), were purchased from Larodan AB (Sweden) and Shanghai ZZBIO (China), respectively. ProElut Silica (1 g/6 ml) gel bonded cartridges were purchased from Dikma Technologies (United States).

### 2.2 Samples collection

Human milk samples were collected from healthy Chinese women. All the volunteers provided three samples including colostrum of 0–5 d, transitional milk of 10–15 d and mature milk of 30 d. All the volunteers had normal physical indicators whose infants were delivered at full term (38–42 weeks of gestation) without congenital or genetic disease. The milk sample was collected from one breast at morning according to a standardized sample collection instruction. The collected human milk was stored at the -80°C refrigerator for later use. The study was approved by Ethics Committee of Beijing Ditan Hospital affiliated to Capital Medical University (#2015-027-01), and all the subjects signed on the informed consent form. The study was also registered at clinicaltrials.gov (with Registration No: NCT02658500).

Bovine milk, goat milk, camel milk, horse milk, and donkey milk were purchased from producers in Beijing, Dalian, Alxa, Urumqi and Jinan, respectively. Infant formulae were purchased from a supermarket in Beijing.

### 2.3 Extraction of lipids from human milk

The lipid extraction procedure was established according to the Bligh & Dyer method (Bligh and Dyer, 1959) with minor modifications. The standards were dissolved to appropriate concentrations with methanol/dichloromethane (1:1, v/v) solution of 5 mM ammonium acetate. Infant formula (1.0 g) was dissolved in ultrapure water (9.0 ml) before extraction. The 200 µL of sample was mixed with the internal standards of 20 μL PC 14:1/17:0 (10.38 mg/L), 20 μL PE 14:1/17:0 (9.64 mg/L), 20 µL SM d17:0/18:1 (10.00 mg/L), 8 µL PI 14:1/17:0 (10.22 mg/L), 4 μL PG 14:1/17:0 (9.81 mg/L), 4 µL Cer d18:2/24:0 (10.00 mg/L) and 20 µL d5-TAG 18:1/18:1/18:1 (4000 mg/L).

First LLE: The mixture mixed with 200 μL ultrapure water, 2 ml methanol and 900 μL dichloromethane were subjected to vortex mixing for seconds, and then 200 μL ultrapure water and 900 μL dichloromethane were added and vortex mixed again. The mixture was centrifuged at 6000 rpm for 15 min to separate the organic phase and aqueous phases.

Second LLE: The aqueous phase extracted by first LLE was mixed with 1.8 ml dichloromethane and centrifuged at 6000 rpm for 15 min to separate the organic phase and aqueous phases.

Third LLE: The organic phase extracted by first LLE was mixed with 1 ml ultrapure water, 2.2 ml methanol and 600 μL dichloromethane and centrifuged at 3000×g for 10 min to separate the organic phase and aqueous phase.

The organic phases obtained from the second and third LLE, as well as the aqueous phase obtained from the second and third LLE were then combined and blown to dry with nitrogen, respectively. The samples from the dried aqueous phase were redissolved in 200 μL methanol/ultrapure water (4:1, v/v) solution for gangliosides detection. And the samples from the dried organic phase were redissolved in 1 ml of methanol/dichloromethane (1:1, v/v) solution of 5 mM ammonium acetate. 50 μL reconstitution sample were diluted 200 times with methanol/dichloromethane (1:1, v/v) solution of 5 mM ammonium acetate for direct detection of glycerides. The other 950 μL reconstitution sample were extracted by solid phase to obtain polar lipids (Ali et al., 2017) as the following.

Firstly, the solid phase (silica cartridges) was activated with 3 ml n-hexane, then 950 μL reconstitution sample was poured into the cartridges, and kept still for 5 min. The non-polar lipids were eluted by 3 ml of hexane/diethyl-ether (8:2, v/v) and 3 ml of hexane/diethyl-ether (1:1, v/v). The polar lipids were eluted with 4 ml of methanol and 4 ml of chloroform/methanol/ultrapure water (3:15:2, v/v/v). The eluted liquid were blown to dry with nitrogen, and redissolved in 190 μL methanol/dichloromethane (1:1, v/v) solution of 5 mM ammonium acetate for the analysis of phospholipids.

### 2.4 Separation conditions by liquid chromatography

The human milk phospholipids were separated on a Kinetex C18 column (Phenomenex, 4.6 mm × 150 mm, 2.6 μm). The injection volume of 2 μL, and the column temperature of 30°C were adopted as the chromatographic conditions. The water/methanol/acetonitrile (v/v/v, 1:1:1) solution of 5 mM ammonium acetate was used as mobile phase A, and the isopropanol solution of 5 mM ammonium acetate was used as mobile phase B. The flow rate was 0.8 ml/min within a total run time of 20 min. The elution gradient was as follows: 0 min, 50% B; 1.0 min, 98% B; 10.0 min,98% B; 15.0 min, 50% B; 20 min, 50% B.

The human milk glycerides were separated on a Kinetex C18 column (Phenomenex, 3 mm × 50 mm, 2.6 μm). The injection volume of 2 μL, and the column temperature of 50°C were adopted as the chromatographic conditions. The water/methanol/acetonitrile (v:v:v, 1:1:1) solution of 5 mM ammonium acetate was used as mobile phase A, and the isopropanol solution of 5 mM ammonium acetate was used as mobile phase B. The flow rate was 0.3 ml/min within a total run time of 10 min. The elution gradient was as follows: 0 min, 50% B; 5.0 min, 98% B; 8.0 min,98% B; 8.01 min, 50% B; 10 min, 50% B.

The human milk gangliosides were separated on a BEH C18 column (Waters, 2.1 mm × 100 mm, 1.7 μm). The injection volume of 10 μL, and the column temperature of 50°C were adopted as the chromatographic conditions. The methanol/water (v/v, 1:9) solution of 1 mM ammonium acetate was used as mobile phase A, and the methanol solution of 1 mM ammonium acetate was used as mobile phase B. The flow rate was 0.3 ml/min within a total run time of 14 min. The elution gradient was as follows: 0 min, 90% B; 0.13 min, 90% B; 3.13 min, 95% B; 4.13 min, 95% B; 6.27 min, 100% B; 12.27 min, 100% B; 12.40 min, 90% B; 14.00 min, 90% B.

### 2.5 Detection conditions of Q-TOF mass spectrometry

The Triple TOF 6600 instrument of AB SCIEX Company was used for detection of human milk phospholipids, glycerides and free fatty acids with ESI source in positive and negative ion modes. MS and MS/MS data were scanned in the range of 50–1300 m/z. The spray voltage of ion source was 5500 V, the declustering potential was 80 V, and the collision energy was 10 V in positive ion mode. And the spray voltage of ion source was −4500 V, the declustering potential was −80 V, and the collision energy was −10 V in negative ion mode. The gas pressure of ion source was 60 psi, the curtain gas pressure was 35 psi and the temperature of ion source was 650°C in both positive and negative ion modes.

The Q-TOF-MS instrument was used for detection of human milk gangliosides with ESI source in negative ion modes. MS and MS/MS data were scanned in the range of 100–2000 m/z. The spray voltage of ion source was −4500 V, the declustering potential was −40 V, and the collision energy was −40 V. The gas pressure of ion source was 40 psi, the curtain gas pressure was 15 psi and the temperature of ion source was 400°C.

### 2.6 Data analysis

The data collected by the Analyst 1.7.1 software were qualitatively analyzed by PeakView 2.2 software according to the fragments of lipids and were quantitatively analyzed by MultiQuant 3.0.2.

The methods for both internal and external standards were used for accurate quantification of phospholipids and glycerides in this experiment. Calibration curves were plotted with the gradient external standards as the abscissa and the ratio between the corresponding areas of external and internal standards as the ordinate. The peak areas of the internal standards, human milk phospholipids and human milk glycerides were integrated from extracted ion chromatograms (EICs). Each lipid class was quantified by one corresponded pair internal-external standard The final concentrations of lipids were expressed in mean ± standard deviations (mg/L).

### 2.7 Method validation

Human milk, and external and internal standards of lipids were used to determine the accuracy, precision, sensitivity, and linearity of the method to validate the method. The limit of detection (LOD) and the linear range of phospholipids and glycerides were determined using internal and external standards. The sensitivity was measured using LOD for each lipid from triplicate measurements. A series of eight dilutions were prepared for phospholipids and glycerides. A gradient series of external and internal standards from 0.001 to 5 mg/L were analyzed in triplicate to evaluate the linearity.

The sample without external standards was set as matrix and the recovery of added external standards was studied at the content similar to the sample. All of the samples need to spike internal standard whose concentrations were written on 2.3.1 and six parallel samples were determined. Three specific concentration levels of external standards of each lipid were as follows: PC, PE: 5 mg/L, 2.5 mg/L, 1.25 mg/L, PI: 0.8 mg/L, 0.4 mg/L, 0.2 mg/L, PG, Cer: 0.4 mg/L, 0.2 mg/L, 0.1 mg/L, SM: 2 mg/L, 1 mg/L, 0.5 mg/L, OPO: 8000 mg/L, 4000 mg/L, 2000 mg/L. The accuracy was evaluated from recovery rate (%). Accordingly, the recovery rate was calculated from the peak area obtained. The precision was estimated by determining low, medium, and high concentrations of external standards of lipids, which was calculated as the relative standard deviation (RSD) of each lipid from six injections. The repeatability of the method was determined by repeated analysis of human milk quality control samples in each batch of analysis carried out on different days over the course of this study. Three parallel samples were prepared for the investigation of repeatability among 3 days.

## 3 Results

### 3.1 Optimization of extracting solvents of lipids

Traditional lipid extraction method is mostly based on LLE introduced by Folch et al. (1957) and modified by Bligh and Dyer (Bligh and Dyer, 1959; Garwolinska et al., 2017), which requires substantially large volumes of toxic organic solvents such as chloroform. In this method, lipids was extract from human milk with dichloromethane instead of chloroform, because dichloromethane was less toxic than chloroform (Wernke and Schell, 2004).

The extraction procedure used in the current study was selected by comparing the results in the species number of extracted lipids. Different lipid extraction solvents were tested in the experiment, and each assay was performed in triplicate. Table 1 shows the species number of lipids in human milk extracted by dichloromethane and chloroform, respectively. Compared with chloroform, dichloromethane could extract more species of human milk lipids.

**TABLE 1:** Species numbers of human milk lipids extracted by different extraction solvents.

	Dichloromethane	Chloroform
PC	14 (including 1 LPC)	6 (including 3 LPC)
PE	25 (including 4 LPE)	12 (including 5 LPE)
PI	11	5 (including 1 LPI)
PS	13 (including 1 LPS)	1
PG	7 (including 1 LPG)	4 (including 2 LPG)
PA	3	6 (including 2 LPA)
SM	14	24
Cer	5	5
DAG	17	16
TAG	107	92
FFA	23	18
Total	239	189

In terms of the number of lipid species, dichloromethane could extract more species of phospholipids (except PA), glycerides and free fatty acids, while chloroform was good at extracting lysophospholipids and sphingolipids. Chloroform only extracted three unsaturated phospholipids, which accounted for 8.8% in the species amount of total glycerophospholipids. High-content glycerophospholipids such as PC 36:2, PE 36:2 and PI 36:2 (Zhao et al., 2021) were not extracted by chloroform. Unsaturated fatty acids, especially polyunsaturated fatty acids, play a crucial role in infant growth, and the development of brain and vision (Glaser et al., 2015; Kevin et al., 2016). Most of the phospholipids in human milk contain unsaturated fatty acids (Lee et al., 2011; Ali et al., 2017; Ingvordsen Lindahl et al., 2019). If a few species of unsaturated phospholipids can be detected by chloroform, it will affect the qualitative and quantitative analysis of phospholipids in human milk. As an extraction solvent, dichloromethane not only can extract more species of lipids, but also has lower toxicity than chloroform. Therefore, dichloromethane is chosen to extract lipids from human milk.

### 3.2 Identification of lipids

**FIGURE 1:**
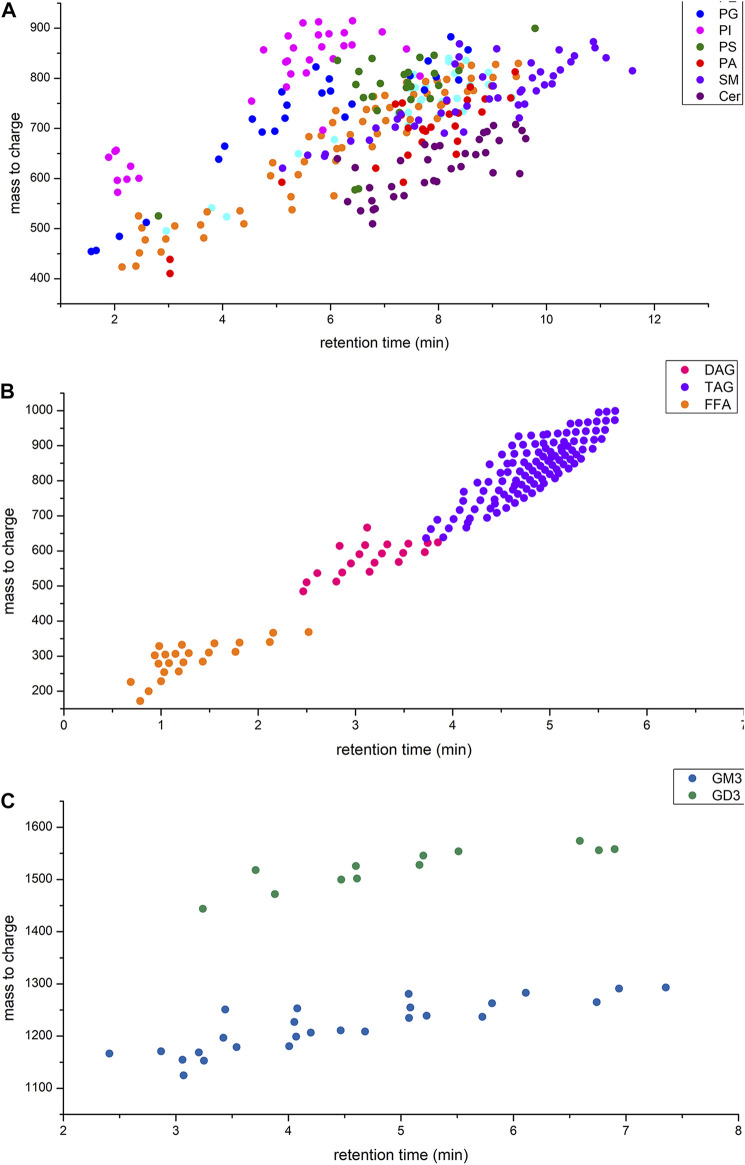
**(A)** Retention time and mass to charge ratios of phospholipids in human milk; **(B)** Retention time and mass to charge ratios of glycerides and free fatty acids in human milk; **(C)** Retention time and mass to charge ratios of gangliosides in human milk.

Various informative fragments in MS/MS mode can be used to differentiate lipid classes and species. Due to the structural diversity of lipids, it is necessary to combine positive and negative ion modes to achieve a comprehensive and clear identification. The optimum instrument detection conditions of human milk phospholipids were decided by MS/MS fragment. Figure 1 shows the mass to charge ratios and retention time range of each lipid class in human milk.

tThe glycerides were identified in positive ion mode with the adduct ion of “+NH_4_”, and the produced fragments included [M − R_1_] ^+^, [M − R_2_]+and [M − R_3_]+in MS/MS. For DAG, one fatty acyl was replaced by oxhydryl. The fatty acyl composition could be calculated by subtracting the fragments from glycerides, respectively (Supplementary Figure S1). Free fatty acid could be directly identified under negative ion mode with [M – H] ^–^ fragment (Supplementary Figure S1). Under the negative ion mode, the adduct ion of PE, PG, PI, PS and PA was “-H^+^” and that of PC was “+CH3COO^−^“. The adduct ion of sphingolipids was “+H^+^” under positive ion mode (Table 2). For PE and PS, the fragment of fatty acyl was produced under negative ion mode and the fragment of phospholipids residue losing a head group was identified in positive ion mode (Supplementary Figures S2, S3). MS/MS spectra of PI, PG, PA demonstrated abundant ions corresponding to the loss of fatty acyl [R_1_]-and [R_2_]-under negative ion mode but no characteristic fragment under positive ion mode (Supplementary Figures S4–6). Therefore, PE, PG, PI, PS and PA were identified under negative ion mode so that the fatty acids composition could be identified easily. For PC and SM containing choline, the phosphocholine fragment ion m/z 184 was produced under positive ion mode (Supplementary Figures S7, S8). Under negative ion mode, the characteristic fragments of PC lost fatty acyl [R_1_]-and [R_2_] ^-^, however, no characteristic fragment of SM was observed. The LCB-2H_2_O (long chain base) ion in positive ion mode (+H^+^) and [R_1_]-in negative ion mode (-H^+^) was allowed for the characterization of detailed structure of Cer (Supplementary Figure S9). The extracted ion chromatogram (XIC) had better base line and chromatographic peak in negative mode, but the fatty acid anions intensity is low. The characteristic fragment ion m/z 264 or 266 showed that the corresponding LCB-2H_2_O included oleic acid (C18:1) or stearic acid (C18:0), respectively.

**TABLE 2:** Characteristic fragments of lipids by UPLC-Q-TOF-MS.

Lipid	Mode	Precursor Ion	Characteristic Fragment
PC	ESI^-^	[M + CH3COO]^-^	[R_1_-H]^-^ [R_2_-H]^-^
PE	ESI^-^	[M-H]^-^	[R_1_-H]^-^ [R_2_-H]^-^
PI	ESI^-^	[M-H]^-^	[R_1_-H]^-^ [R_2_-H]^-^
PS	ESI^-^	[M-H]^-^	[R_1_-H]^-^ [R_2_-H]^-^
PG	ESI^-^	[M-H]^-^	[R_1_-H]^-^ [R_2_-H]^-^
PA	ESI^-^	[M-H]^-^	[R_1_-H]^-^ [R_2_-H]^-^
SM	ESI^+^	[M + H]^-^	184.07
Cer	ESI^+^	[M + H]^-^	264.26/266.28
DAG	ESI^+^	[M + NH4] ^+^	[M-R_1_]+[M-R_2_] ^+^
TAG	ESI^+^	[M + NH4] ^+^	[M- R_1_]+[M- R_2_]+[M- R_3_] ^+^
FFA	ESI^-^	[M-H]^-^	[M-H]^-^
GM3	ESI^-^	[M-H]^-^	290.08
GD3	ESI^-^	[M-2H]^2-^	290.08

For phospholipids, the characteristic fragments of sphingolipids and glycerophospholipids need to be collected in positive and negative ion mode, respectively. Thus, the chromatographic method was established based on two consecutive separations of phospholipids by LC. The method was used in the positive ion mode for the first run followed by a data dependent MS-MS analysis, and then the same chromatography method was used for reanalysis in the negative ion mode for the second run. Glycerides (positive ion mode) and free fatty acids (negative ion mode) were detected in the same way as phospholipids.

The gangliosides were identified in negative ion mode, the adduct ion of GM3 was “-H” and GD3 was “-2H ^2-^“. And sialic acid fragment ion m/z 290 was produced as the characteristic fragment of GM3 and GD3 (Supplementary Figure S10).

All in all, lipids qualitative analysis used m/z with adduct ion to extract secondary mass spectra from TIC and the corresponding characteristic fragments were used to identified lipids species. The typical extracted ion chromatogram of lipids were shown in Supplementary Figure S11. The characteristic fragments of each class of lipids were shown in Table 2. Although some lipids that mass to charge were similar (Figure 1), they have the different headgroup or fatty acids composition. Thus, this qualitative analysis method could distinguish lipids species clearly and ensure the reliability of results.

### 3.3 Method validation

According to the contents of various lipids in human milk, the external standards of different concentrations were prepared, a gradient series of external and internal standards were analyzed in triplicate to evaluate the linearity. The standard curves were plotted with the gradient concentration of external standards as the abscissa and the corresponding area ratio between external and internal standards as the ordinate. The regression equations and coefficient of determination R^2^ are shown in Table 3. Among the concentration range of phospholipids and glycerides, the correlation coefficients are all above 0.99 within the working range. LOD is defined as the smallest amount of a lipid that could be identified from the characteristic fragments required for qualitative analysis. The LODs for most lipids were well below 0.001 mg/L (Table 3), demonstrating high sensitivity of this method.

**TABLE 3:** LOD, LOQ, linearity of lipid standards and corresponding internal standards.

Standard	Internal standard	LOD (mg/L)	Calibration equations	R^2^	Range (mg/L)
PC (18:0/18:2)	PC (14:1/17:0)	0.0006	Y = 1.0891X+0.0064	0.9995	0.0025–5
PE (18:0/18:2)	PE (14:1/17:0)	0.0005	Y = 1.7927X-0.0274	0.9993	0.0025–5
PI (16:0/18:1)	PI (14:1/17:0)	0.0005	Y = 6.0527X-0.3599	0.9986	0.001–1
PG (16:0/18:1)	PG (14:1/17:0)	0.0003	Y = 7.9927X-0.1704	0.9995	0.001–1
SM (d18:1/24:0)	SM (d18:1/17:0)	0.0005	Y = 0.6395X-0.0018	0.9980	0.0025–5
Cer (d18:1/24:1)	Cer (d18:2/24:0)	0.0008	Y = 3.7307X-0.0617	0.9927	0.001–1
TAG (16:0/18:1/18:1)	TAG (16:0/18:1/18:1 (d5))	0.0001	Y = 7.5236X+0.1681	0.9993	0.0025–5

Human milk sample was used as a matrix in the experiment to measure recovery. It was determined that the spiked samples of three-level standards were within the working range. Three specific concentration levels of external standards of each lipid were determined 6 times. The average recovery rate of various lipids was 79.21%–117.98%. The experimental results (Table 4) showed that the analytical method could meet the quantitative detection requirements. The precision of the method was evaluated using the mixed lipid standards. Mixed lipid standards were prepared and determined 6 times during the day and night, respectively. The relative standard deviation (RSD) of the lipids concentration was 1.98%–9.80%. It could be known from the result shown in Table 4 that the instrument method had good precision. Through the detection of the same human milk sample for 3 days, it could be seen that the RSD of this method was 6.35%–12.89%, which was less than 15% and could meet the requirement of mass spectrometry repeatability.

**TABLE 4:** Repeatability (RSD, %) of standards and sample.

Standard	Internal standard	Standard addition concentration	Recovery%	Repeatability%	RSD% (day)	RSD% (night)
PC (18:0/18:2)	PC (14:1/17:0)	5	97.1	9.39	2.86	3.57
		2.5	93.17		4.31	7.63
		1.25	113.55		7.20	9.43
PE (18:0/18:2)	PE (14:1/17:0)	5	102.1	6.35	8.92	7.07
		2.5	90.38		5.13	2.02
		1.25	117.98		7.47	3.76
PI (16:0/18:1)	PI (14:1/17:0)	0.8	95.3	8.88	7.58	6.85
		0.4	84.79		5.41	4.62
		0.2	102.53		8.91	4.28
PG (16:0/18:1)	PG (14:1/17:0)	0.4	102.6	10.68	6.46	1.98
		0.2	86.32		7.68	2.45
		0.1	108.35		6.58	2.19
SM (d18:1/24:0)	SM (d18:1/17:0)	2	91.1	11.51	7.46	9.26
		1	79.21		7.05	7.05
		0.5	115.07		8.46	8.10
Cer (d18:1/24:1)	Cer (d18:2/24:0)	0.4	94.7	12.89	7.03	6.24
		0.2	82.65		9.80	8.96
		0.1	80.04		9.21	6.39
TAG (16:0/18:1/18:1)	TAG (16:0/18:1/18:1 (d5))	8000	94.5	10.87	5.51	4.66
		4000	81.51		5.68	5.83
		2000	104.54		7.84	9.21

### 3.4 Application of the method in human milk, other mammalian milk, and infant formulae

**FIGURE 2:**
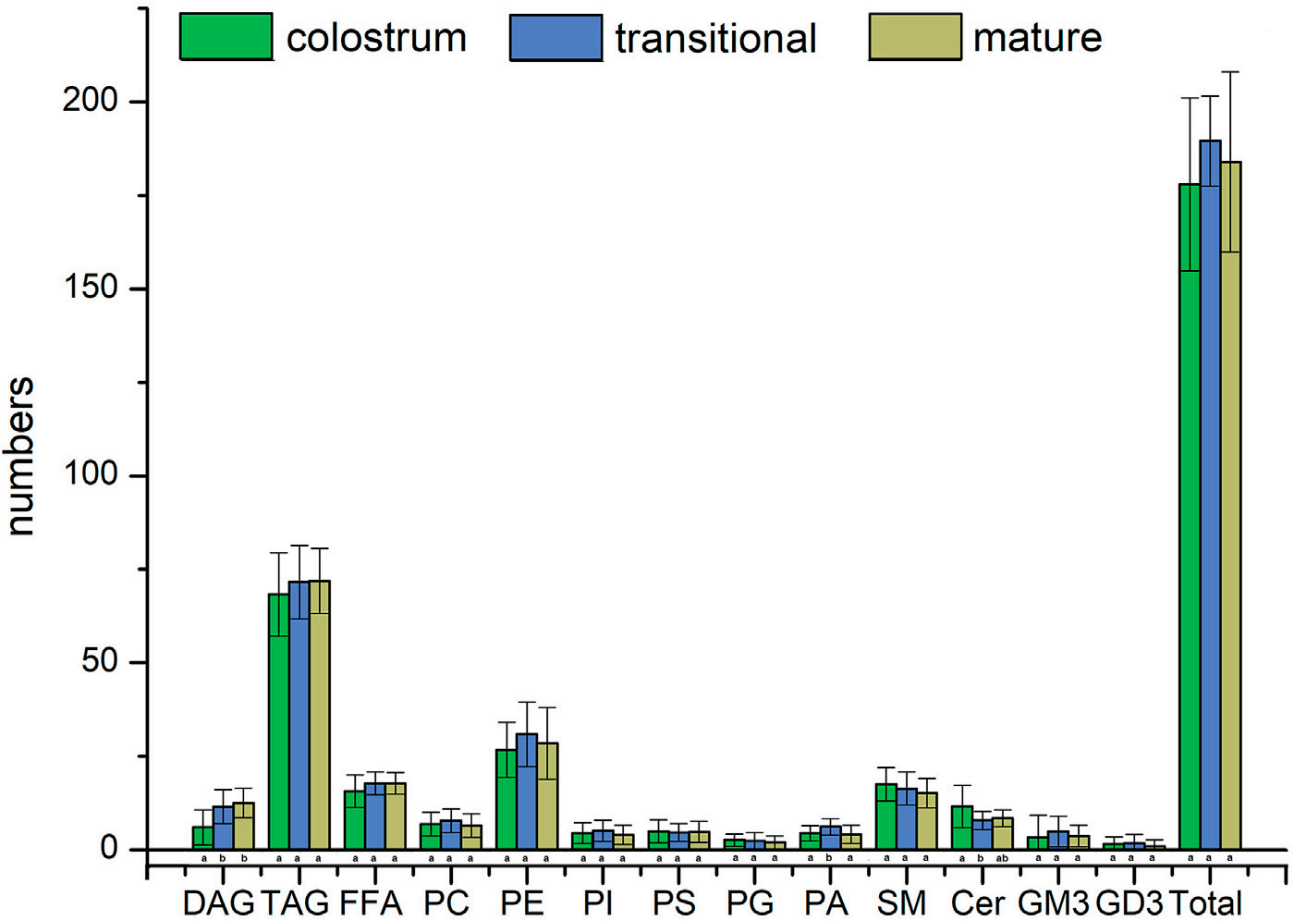
Species number of human milk lipids at different lactation stages.

**FIGURE 3:**
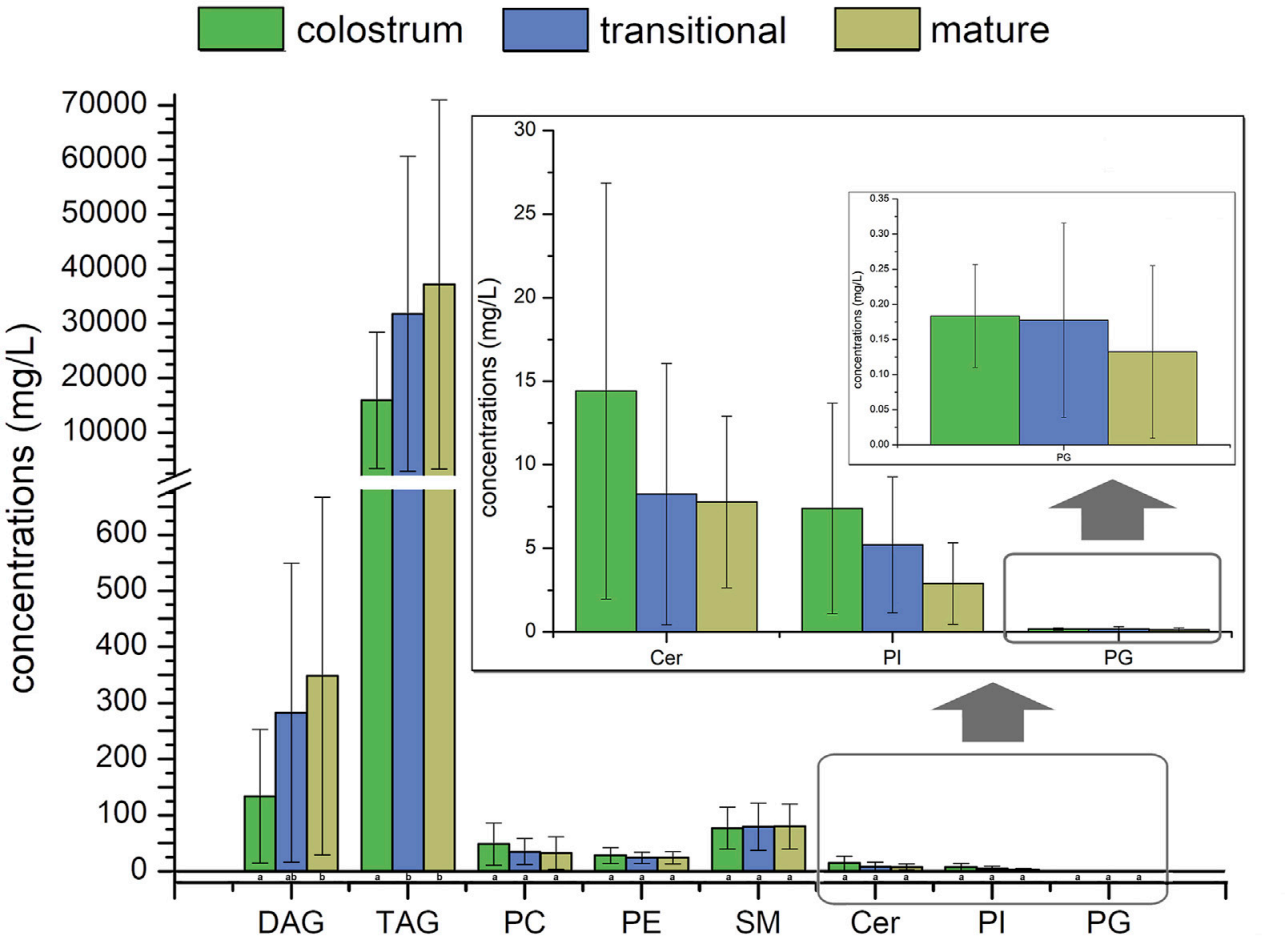
Concentrations of human milk lipids at different lactation stages.

Figure 2; Figure 3 showed the result of 57 human milk samples, including colostrum, transitional milk and mature milk. The species of phospholipids, glycerides, free fatty acids and gangliosides were qualitatively analyzed, and the concentrations of phospholipids, glycerides were absolutely quantitatively analyzed by internal standard method.

In the human milk, there were totally 483 species of lipids, including 21 species of PC, 60 species of PE, 18 species of PI, 21 species of PS, 20 species of PG, 24 species of PA, 46 species of SM, 35 species of Cer, 29 species of DAG, 148 species of TAG, 23 species of FFA, 26 species of GM3 and 12 species of GD3. The list of lipids identified in human milk were shown in Supplementary Tables S1–3. In the transitional milk, the species of total lipids were the most abundant. Among three lactation stages, the species number of glycerides in mature milk was the most, the species number of PC, PE, PI, and PA in transition milk was the most, the species number of PG, SM, and Cer in colostrum milk was the most, and the species number of PS was basically stable in three lactation stages. The species of DAG in transitional milk and mature milk were significantly higher than those in colostrum, and the species of PA in transitional milk were significantly lower than those in colostrum and mature milk. There was no significant difference between the species of Cer in transitional milk and those in mature milk, but the species of Cer in colostrum were significantly higher than those in mature milk.

The internal standards of PS, PA, FFA, GD3 and GM3 were not added; therefore, they were not accurately quantified in this study. The concentrations of TAG were higher than those of other lipids in human milk, it accounted for more than 98% of the lipids measured, with concentration significantly increased from 15441.6 mg/L in colostrum to 35996.3 mg/L in mature milk. Meanwhile, the concentrations of SM were higher than that of other phospholipids in human milk, accounting for more than 44% of the phospholipids measured, with concentration increased from 76.9 mg/L in colostrum to 79.7 mg/L in mature milk. In addition, the concentrations of DAG increased with the lactation phase, and the concentrations of other phospholipids decreased with the lactation phase.

**FIGURE 4:**
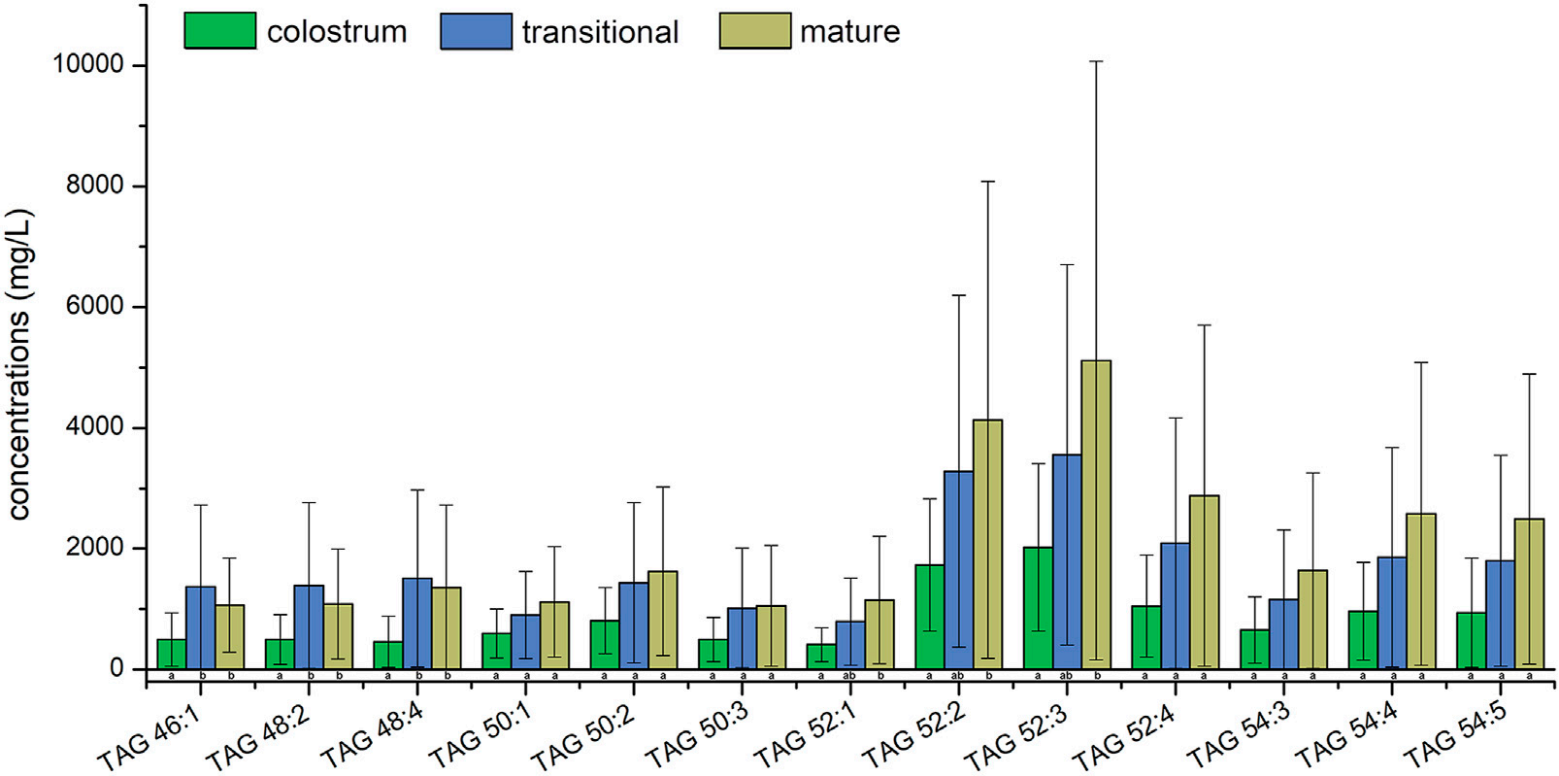
Average concentrations of top ten human milk glycerides at different lactation stages.

**FIGURE 5:**
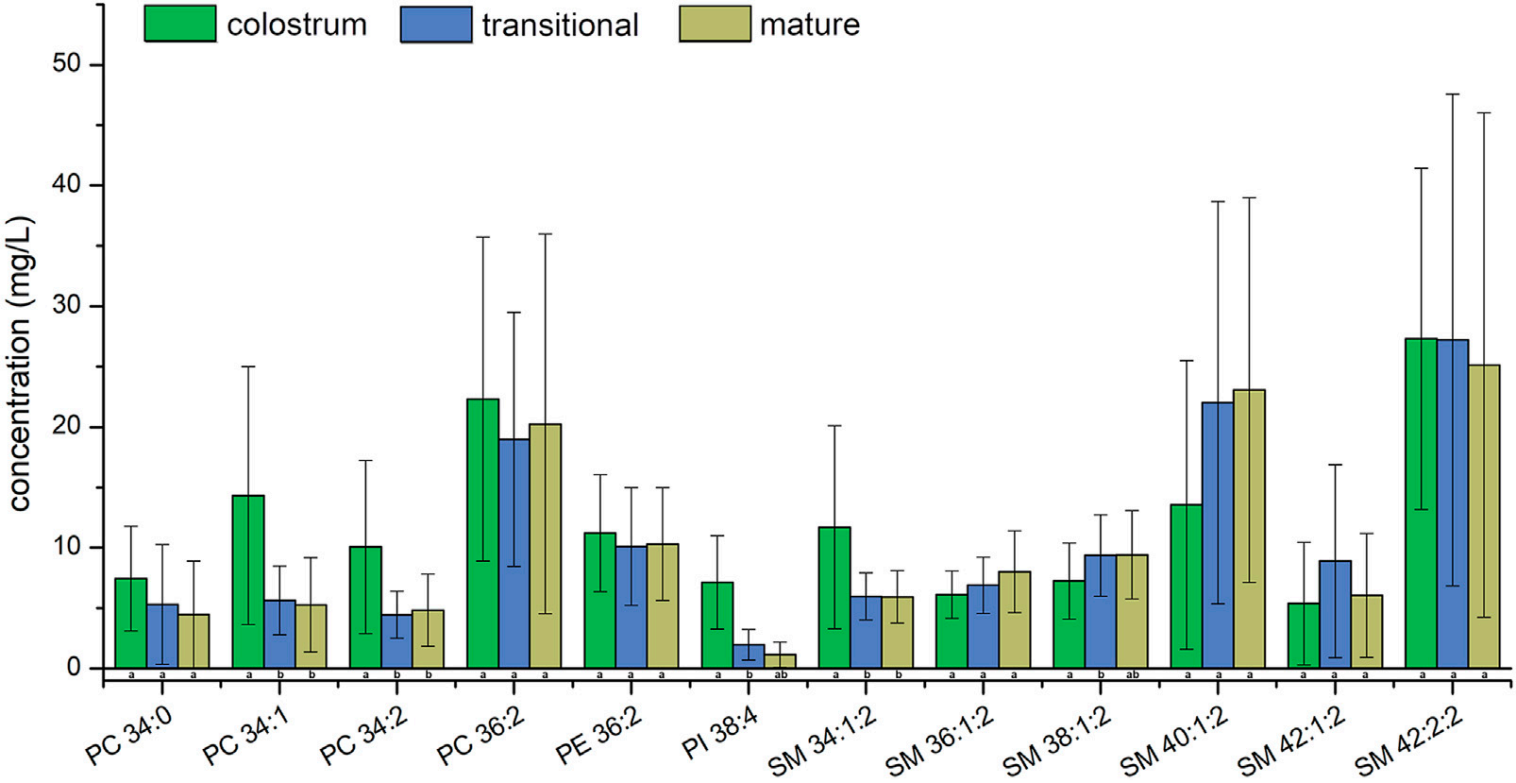
Average concentrations of top ten human milk phospholipids at different lactation stages.

Top ten lipids of average concentration in three stages were shown in Figure 4; Figure 5, the species of top ten glycerides and phospholipids were a bit different among three stages. Only four TAGs, including TAG 48:4, TAG 50:2, TAG 52:2, and TAG 54:4 was detected in all of the human milk samples. The glycerides of TAG52:3, TAG52:2, TAG 52:4, and TAG 54:4 had the highest concentrations which totally accounted for more than 31.6% in all glycerides and increased gradually among three stages. The concentrations of TAG 46:1, TAG 48:2, and TAG 48:4 in transitional milk and mature milk were significantly higher than those in colostrum. There was no significant difference between the concentrations of TAG 52:1, TAG 52:2, and TAG 54:4 in mature milk and those in transitional milk, but the concentration of those in mature milk were significantly higher than those in colostrum.

The concentrations of PC 36:2, PE 36:2 and SM 42:2;2 were the highest among all PC, PE and SM in three stages, respectively. Although the concentrations of PI 38:4 was highest among PIs molecular species in colostrum, it was the second highest in transitional milk and mature milk while that of PI 36:2 was the highest. Only SM 34:1;2 and SM 42:2;2 were detected in all of the human milk samples. The phospholipids of SM 42:2;2 had the highest concentration which accounted for more than 11.1% in all phospholipids among three stages and the proportion of the top ten lipids in total lipids gradually increased. The concentrations of PC 34:1, PC 34:2, and SM 34:1;2 in transitional milk and mature milk were significantly lower than those in colostrum. There was no significant difference between the concentrations of PI 38:4 and SM 38:1;2 in mature milk and those in transitional milk, but the concentrations of those in transitional milk were significantly lower than those in colostrum.

Among the top ten concentration PIs, PI 38:4 is rich in arachidonic acid (AA, C20:4), which is more abundant in colostrum. AA is critical for growth, brain development, and health of infants (Kevin et al., 2016), therefore, PI 38:4 may be an important source of AA in phospholipids from human milk. Linoleic acid (C18:2) is an essential fatty acid existing in TAG 50:3, TAG 52:3, TAG 52:4, TAG 54:4, TAG 54:5, PC 34:2, PC 36:2, and PE 36:2 with high concentration.

The species number of lipids in human milk were more abundant than that in other mammalian milk and infant formula (Table 5). Other mammalian milk and infant formula had more than 200 species of lipids, including phospholipids, glycerides, free fatty acids and gangliosides. However, there were only 124 species of lipids in donkey milk.

**TABLE 5:** The total species number of lipids in other mammalian milk and infant formula.

Lipids	Human milk	Bovine milk	Goat milk	Camel milk	Horse milk	Donkey milk	Infant formula
PC	21	13	8	10	10	4	13
PE	60	27	29	28	34	17	25
PI	18	17	12	12	15	7	17
PS	21	8	7	9	5	2	6
PG	20	8	3	7	8	0	6
PA	24	8	6	7	7	1	5
SM	46	25	17	17	15	21	25
Cer	35	7	7	9	5	6	8
DAG	29	10	3	4	2	0	13
TAG	148	117	97	92	94	57	115
FFA	23	8	11	8	9	9	5
GM3	26	0	1	7	0	0	0
GD3	12	9	3	0	0	0	5
Total	483	249	204	209	204	124	243

**FIGURE 6:**
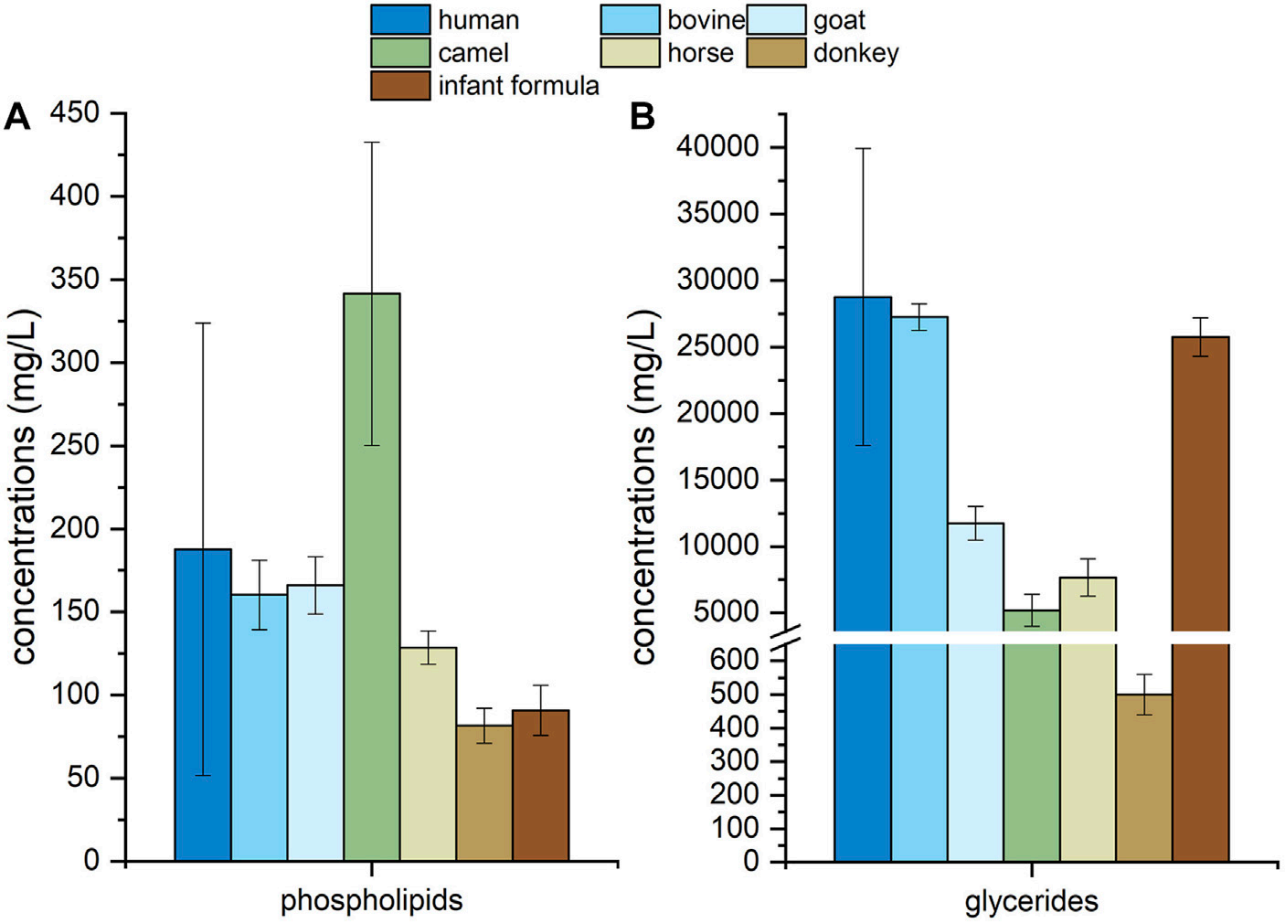
Concentrations of phospholipids **(A)** and glycerides **(B)** in human milk, mammalian milk and infant formula.

For human milk, other mammalian milk and infant formula, totally 556 species of lipids were detected, including 29 species of PC, 61 species of PE, 27 species of PI, 32 species of PS, 25 species of PG, 28 species of PA, 46 species of SM, 35 species of Cer, 30 species of DAG, 176 species of TAG, 23 species of FFA, 26 species of GM3 and 18 species of GD3. 68 species of lipids existing in other mammalian milk or infant formulae were not detected in human milk. Total concentrations of phospholipids and total glycerides in human milk, other mammalian milk and infant formula were shown in Figure 6. Compared with those in the majority of other mammalian milk and infant formula, the concentration of total phospholipids and total glycerides were present in greater amounts in human milk. The concentration of total phospholipids in camel milk were highest among these samples while those of total phospholipids and total glycerides in donkey milk were lowest.

## 4 Discussion

Human milk lipids provide energy for the growth and development of infants (Tingting, 2008). Different molecular components of glycerides and phospholipids in human milk are closely related to their physiological functions (Huang et al., 2013; Zhao et al., 2021). Therefore, an accurate and reliable method is essential for a comprehensive identification and absolute quantitation of lipids at molecular level. The extraction, separation, identification and quantification methods of lipids from human milk and dairy products are shown in Table 6.

**TABLE 6:** Comparison of extraction and analytical methods used for lipids in milk and dairy products.

Sample	Extraction Method	Instrumentation	Lipids Identified	Number of Lipids	Quantification	References
Human milk	LLE, chloroform/methanol (2:1, v/v), 1 times extraction	UPLC-Q-TOF-MS	PC, PE, PI, PS, SM, TAG	136	Relative quantification	Sokol et al. (2015)
Human milk	LLE, dichloromethane/methanol (2:1, v/v), 3 times extraction	UPLC-APCI- MS	TAG	42	Relative quantification	Ten-Domenech et al. (2015)
Human milk, formula milk, bovine milk	SPME, methanol: H_2_O (5:1, v/v)	UPLC-ESI-Q-TOF-MS	PC, PE, PI, PS, PG, PA, SM, Cer, DAG, TAG, FFA, PR, ST	764	Cannot quantification	Garwolinska et al. (2017)
Bovine milk, goat milk, soymilk	LLE, chloroform/methanol (2:1, v/v), 1 times extraction	UPLC-Q-Exactive Orbitrap MS	PC, PE, PI, PS, PG, PA, SM, Cer, DAG, TAG, FFA	462	Absolute quantification	Li et al. (2017)
Human milk	LLE, n-hexane, 1 times extraction	SFC-Q-TOF-MS	DAG, TAG	95	Relative quantification	Tu et al. (2017)
Human milk, infant formula, butter milk powder	LLE, chloroform/methanol (2:1, v/v), 2 times extraction	HPLC-ESI-MS	PC, PE, PI, Cer	62	Absolute quantification	Tavazzi et al. (2018)
Human milk, infant formula	LLE, chloroform/methanol (2:1, v/v), 2 times extraction	HPLC-ESI-IT-TOF-MS	PC, PE, PI, PS, PG, PA, SM	161	Absolute quantification	Jiang C. et al. (2018)
Bovine milk	LLE, MTBE: methanol (5:1, v/v), 3 times extraction	UPLC-ESI-Q-TOF-MS	PC, PE, PI, PS, PG, PA, SM, Cer, DAG, TAG, HexCer, Hex2Cer, CL	335	Absolute quantification	Li et al. (2020)
Bovine milk	LLE, chloroform/methanol (2:1, v/v), 1 times extraction	UPLC-HESI-Q Exactive-MS	PC, PE, PI, PS, PG, PA, PC-P, PE-P, SM, Cer, GluCer, LacCer, GD3, GM3	524	Absolute quantification	Liu et al. (2020)
Infant formula, growing up milk powders, raw materials	PLs: LLE, chloroform/methanol (9/1, v/v), 2 times extraction	PLs: HPLC-ELSD	PC, PE, PI, PS, SM, GD3, GM3	No mentioned	Absolute quantification	Moloney et al. (2020)
	GAs: LLE & SPE, chloroform/methanol, 2 times extraction	GAs: UPLC-Q-TOF-MS				
Human milk, bovine milk, caprine milk	LLE, MTBE/methanol (5:1, v/v), 3 times extraction	UPLC-ESI-Q-TOF-MS	PC, PE, PI, PS, PG, PA, SM, Cer, DAG, TAG, HexCer, Hex2Cer, CL	348	Relative quantification	Wang et al. (2020)
Human milk	LLE, chloroform/methanol (2:1, v/v), 1 times extraction	UPLC-ESI-IT-TOF-MS	PC, PE, PS, PI, PG, PA, SM	258	Absolute quantification	Song et al. (2021)
Human milk, infant formulae, other mammalian milk	LLE & SPE, dichloromethane/methanol (1:2, v/v), 3 times extraction	UPLC-ESI-Q-TOF-MS	PC, PE, PI, PS, PG, PA, SM, Cer, DAG, TAG, FFA, GD3, GM3	556	Absolute quantification	This study

Methyl-tert-butyl ether (MTBE).

LLE technique was used to separate lipids by their relative solubilities in different immiscible liquids (George et al., 2018). Extracting solvents and process were key to obtain lipids from samples. In the Folch (Folch et al., 1957) and Bligh–Dyer(Bligh and Dyer, 1959) method, chloroform was commonly used to extract lipids from human milk or dairy products (Sala Vila et al., 2003; Ten-Domenech et al., 2015; Claumarchirant et al., 2016; Tang et al., 2021). When chloroform is exposed to light, it could react with oxygen in the air and is gradually decomposed to produce the highly toxic phosgene, which has anesthetic effect on heart, liver and kidney. The data analysis of human milk lipids requires to process a large number of samples and it might be harmful to the health of the operators who have been engaged in the detection for a long time. Therefore, safety solvents are beneficial for the health of the operator and it is necessary to establish safer and more reliable methods. We extracted lipids for 3 times with dichloromethane instead of chloroform in this study, and obtained aqueous phase containing gangliosides and organic phase containing glycerides and phospholipids. In contrast, most of studies used twice extraction (Jiang C. et al., 2018; Alexandre-Gouabau et al., 2018; Tavazzi et al., 2018; Moloney et al., 2020) or once extraction (Sokol et al., 2015; Li et al., 2017; Liu et al., 2020; Song et al., 2021). However, fewer species of glycerides and phospholipids were obtained and ganglioside was not enriched.

The concentrations of glycerides and phospholipids accounted for about 98% and 0.8% in human milk lipids, respectively (Delplanque et al., 2015; Zou et al., 2017; George et al., 2018), leading to the wide concentration range of different lipid subclasses in human milk. If all lipid classes were quantified simultaneously in one analytical cycle, the MS signal of main glycerides could become saturated, which would not only influence quantitative results, but also contaminate the instrument. Meanwhile, the MS signals for phospholipid and ganglioside classes were very low. Therefore, the organic phase needs to be further processed with solid phase extraction to obtain phospholipids. The silica SPE column, which was usually applied in the separation of polar and non-polar lipids and showed good accuracy (Contarini and Povolo, 2013; Verardo et al., 2017), was used in this method. The obtained phospholipids could be detected without dilution and with high signal intensity.

This method not only reduced the use of hazardous chloroform solvent, but also extracted phospholipids, glycerides and free fatty acids, even gangliosides in an individual process. In contrast, only a few of methods could extract two or more classes of lipids among phospholipids, glycerides, free fatty acids and gangliosides in human milk through one preparation process (Sokol et al., 2015; Garwolinska et al., 2017; Alexandre-Gouabau et al., 2018; Wang et al., 2020). In published studies, gangliosides only could be extracted from human milk individually or together with phospholipids that used more than 250 µL human milk (McJarrow et al., 2019; Liu et al., 2020). Other lipid extraction methods usually extract glycerides, phospholipids and gangliosides, respectively (Zou et al., 2013; Wei et al., 2019a; Wei et al., 2019b), which require more solvents, human milk samples, and long and complex extraction process.

In the present study, glycerophospholipids and sphingolipids were detected in negative mode and positive mode, respectively. In contrast, many studies detected glycerophospholipids and sphingolipids in the same ion mode (Garwolinska et al., 2017; Jiang C. et al., 2018; Song et al., 2018; Ingvordsen Lindahl et al., 2019; Mitina et al., 2020; Garwolinska et al., 2021). Some studies only identified headgroup of glycerophospholipids, the number of carbon atoms and double bonds in total fatty acyls through fragment, without fatty acyls moieties (Garwolinska et al., 2017; Tavazzi et al., 2018). Meanwhile, most studies identified glycerophospholipids through the residue losing a fatty acyl (Donato et al., 2011; Ali et al., 2017; Jiang C. et al., 2018; Liu et al., 2020) for qualitative analysis of glycerophospholipids. In contract, this study could identify the fatty acid composition of glycerophospholipids more easily according to m/z of fragment. Cer and gangliosides could also be detected in negative ion mode, and more lipids classed (HexCer, Hex2Cer) and fatty acid composition were identified by MS/MS and MS^3^ (Calvano et al., 2019). Meanwhile, milk sphingolipids, including (SM and Cer), were detected in positive mode in most of the literature (Liu et al., 2020; Wang et al., 2020; Zhao et al., 2021) and the comparison results of this method shows that the characteristic fragment ion m/z 264 or 266 directly indicates the ceramides long-chain base including C18:1 or C18:0, respectively. In negative mode, although XIC had better chromatographic peak, the characteristic fragment ([R1]^-^) intensity was too low (Supplementary Figure S9) that may influence the accuracy of qualitative analysis, especially for those low concentration Cer. In some studies, Cer species detected in negative mode were less than that in positive mode (Liu et al., 2020; Wang et al., 2020), while some of Cer also was higher level in human milk phospholipids (Alexandre-Gouabau et al., 2018; Zhao et al., 2021). Thus, the positive mode was finally chosen for the detection of Cer.

High-resolution mass spectrum was good at qualitative analysis, while it was a bit instable in quantification. So, if internal standards were not added, the intraassay results of recovery and the reproducibility would be poor. Adding internal standards with similar structure to the existing lipids in human milk is an effective way to eliminate the impact of poor instrument stability for quantitative analysis. Except for d5-TAG 18:1/18:1/18:1, the internal standards of other phospholipids were not deuterated. Meanwhile, PC 14:1/17:0, PE 14:1/17:0, SM d17:0/18:1, PI 14:1/17:0, PG 14:1/17:0, and Cer d18:2/24:0 was not detected in human milk, indicating that the addition of internal standard will not influence the content of the lipids in human milk. The addition of internal standards could ensure the accuracy of quantitative results and the comparability between the corresponding batches.

Due to the high resolution of TOF, the number of lipids species, including phospholipids, glycerides, free fatty acids and gangliosides, in human milk reported in this study were higher compared with that in most previous researches (Li et al., 2017; Wang et al., 2020; Song et al., 2021). It was shown that this method had good performance in the characterization of human milk lipids. Meanwhile, this method could be used for absolute quantification of each species of phospholipids and glycerides, which was beneficial for the omni-directional analysis of the composition and content of lipids in human milk.

Human milk is the golden standard for the development of infant formula. In most infant formulae, other mammalian milk, especially bovine milk, is mixed with vegetable oils in order to mimic the fatty acid profile of human milk (Tu et al., 2017; Jiang T. et al., 2018). It is necessary to profile the lipids in human milk, other mammalian milk and infant formula with the same extraction and detection method. Table 5 showed that infant formula and other mammalian milk had some difference in lipid composition from human milk, while the species number of lipids in the former was all lower than that in human milk. And no gangliosides were detected in horse milk and donkey milk. The concentrations of phospholipids in bovine milk and goat milk were similar to that in human milk. Meanwhile, the concentration of phospholipids in camel milk was higher than that in human milk and infant formula, those of horse milk and donkey milk was lower. The concentration of glycerides in bovine milk, infant formula and human milk were similar, the glycerides concentration of other mammalian milk was much lower. These results suggested that the present method would also be a reliable tool for the profiling of lipids in other mammalian milk and infant formula.

## 5 Conclusion

In this study, a qualitative and quantitative method was established and validated for profiling phospholipids and glycerides in human milk using UPLC-Q-TOF-MS with LLE and SPE. This method could extract a variety of lipids including phospholipids, glycerides, free fatty acids and gangliosides from a small volume of human milk after one series of pretreatment. We applied the established method to analyze a large number of human milk samples and detected 245 species of phospholipids, 177 species of glycerides, 23 species of free fatty acids and 38 species of gangliosides. Meanwhile, the use of internal standard allowed quantitative analysis of lipids and also corrected the loss of analyte during sample preparation and detection. The applicability of this method was validated by analyzing lipids in human milk, other mammalian milk and infant formula.

## Data Availability

The data presented in the study are deposited in Figshare repository, accession DOI: 10.6084/m9.figshare.21785891.v1.

## Supplementary material

Supplementary Figure S1
**(A)** MS/MS fragments of TAG 52:3 acquired in positive ion mode; **(B)** MS/MS fragments of DAG 34:2 acquired in negative ion mode; **(C)** MS/MS fragments of FFA 18:1 acquired in negative ion mode.**(A)** MS/MS fragments of PE 36:2 acquired in positive ion mode; **(B)** MS/MS fragments of PE 36:2 acquired in negative ion mode.

Supplementary Figure S2
**(A)** MS/MS fragments of PE 36:2 acquired in positive ion mode; **(B)** MS/MS fragments of PE 36:2 acquired in negative ion mode.

Supplementary Figure S3
**(A)** MS/MS fragments of PS 36:0 acquired in positive ion mode; **(B)** MS/MS fragments of PS 36:0 acquired in negative ion mode.

Supplementary Figure S4
**(A)** MS/MS fragments of PI 34:1 acquired in positive ion mode; **(B)** MS/MS fragments of PI 34:1 acquired in negative ion mode.

Supplementary Figure S5
**(A)** MS/MS fragments of PG 38:5 acquired in positive ion mode; **(B)** MS/MS fragments of PG 38:5 acquired in negative ion mode.

Supplementary Figure S6
**(A)** MS/MS fragments of PA 34:1 acquired in positive ion mode; **(B)** MS/MS fragments of PA 34:1 acquired in negative ion mode.

Supplementary Figure S7
**(A)** MS/MS fragments of PC 36:2 acquired in positive ion mode; **(B)** MS/MS fragments of PC 36:2 acquired in negative ion mode.

Supplementary Figure S8
**(A)** MS/MS fragments of SM 38:1;2 acquired in positive ion mode; **(B)** MS/MS fragments of SM 38:1;2 acquired in negative ion mode.

Supplementary Figure S9
**(A)** MS/MS fragments of Cer 42:2;2 acquired in positive ion mode; **(B)** MS/MS fragments of Cer 42:2;2 acquired in negative ion mode.

Supplementary Figure S10
**(A)** MS/MS fragments of GM3 36:1;2 acquired in negative ion mode; **(B)** MS/MS fragments of GD3 34:1;2 acquired in negative ion mode.

Supplementary Figure S11
**(A)** Extracted ion chromatogram of SM 38:1;2 acquired in positive ion mode; **(B)** Extracted ion chromatogram of PE 36:2 acquired in negative ion mode; **(C)** Extracted ion chromatogram of TAG 52:3 acquired in positive ion mode; **(D)** Extracted ion chromatogram of FFA 18:1 acquired in negative ion mode; **(E)** Extracted ion chromatogram of GM3 36:1;2 acquired in negative ion mode.

Supplementary Table S1 List of identified phospholipids in human milk detected by UPLC-Q-TOF-MS.

Supplementary Table S2 List of identified glycerides and free fatty acids in human milk detected by UPLC-Q-TOF-MS.

Supplementary Table S3 List of identified gangliosides in human milk detected by UPLC-Q-TOF-MS.

## Abbreviations

PC, phosphatidylcholine; PE, phosphatidylethanolamine; PI, phosphatidylinositol; PG, phosphatidylglycerol; PS, phosphatidylserine; PA, phosphatidic acid; SM, sphingomyelin; Cer, ceramides; LPC, lysophosphatidylcholine; LPE, lysophosphatidylethanolamine; LPI, lysophosphatidylinositol; LPG, lysophosphatidylglycerol; LPS, lysophosphatidylserine; LPA, lysophosphatidic acid; DAG, diacylglycerols; TAG, triacylglycerols; FFA, free fatty acid; UPLC-Q-TOF-MS, Ultra-Performance Liquid Chromatography with Quadrupole-Time-of-Flight Mass Spectrometry; LLE, liquid-liquid extraction; SPE, solid phase extraction.
