# Supplementary material for: Quantification of phospholipids and glycerides in human milk using ultra-performance liquid chromatography with quadrupole-time-of-flight mass spectrometry
Source: Front Chem. 2023 Jan 9;10:1101557. doi: 10.3389/fchem.2022.1101557 (PMC9868747; doi:10.3389/fchem.2022.1101557)
Supplement: Supplementary file 1 [file DataSheet1.docx]

Supplementary Material

[**Figure S1 (A) MS/MS fragments of TAG 52:3 acquired in positive ion mode; (B) MS/MS fragments of DAG 34:2 acquired in negative ion mode; (C) MS/MS fragments of FFA 18:1 acquired in negative ion mode.**](#_Toc121317993)

[**Figure S2 (A) MS/MS fragments of PE 36:2 acquired in positive ion mode; (B) MS/MS fragments of PE 36:2 acquired in negative ion mode.**](#_Toc121317994)

[**Figure S3 (A) MS/MS fragments of PS 36:0 acquired in positive ion mode; (B) MS/MS fragments of PS 36:0 acquired in negative ion mode.**](#_Toc121317995)

[**Figure S4 (A) MS/MS fragments of PI 34:1 acquired in positive ion mode; (B) MS/MS fragments of PI 34:1 acquired in negative ion mode.**](#_Toc121317996)

[**Figure S5 (A) MS/MS fragments of PG 38:5 acquired in positive ion mode; (B) MS/MS fragments of PG 38:5 acquired in negative ion mode.**](#_Toc121317997)

[**Figure S6 (A) MS/MS fragments of PA 34:1 acquired in positive ion mode; (B) MS/MS fragments of PA 34:1 acquired in negative ion mode.**](#_Toc121317998)

[**Figure S7 (A) MS/MS fragments of PC 36:2 acquired in positive ion mode; (B) MS/MS fragments of PC 36:2 acquired in negative ion mode.**](#_Toc121317999)

[**Figure S8 (A) MS/MS fragments of SM 38:1;2 acquired in positive ion mode; (B) MS/MS fragments of SM 38:1;2 acquired in negative ion mode.**](#_Toc121318000)

[**Figure S9 (A) MS/MS fragments of Cer 42:2;2 acquired in positive ion mode; (B) MS/MS fragments of Cer 42:2;2 acquired in negative ion mode.**](#_Toc121318001)

[**Figure S10 (A) MS/MS fragments of GM3 36:1;2 acquired in negative ion mode; (B) MS/MS fragments of GD3 34:1;2 acquired in negative ion mode.**](#_Toc121318002)

[**Figure S11 (A) Extracted ion chromatogram of SM 38:1;2 acquired in positive ion mode; (B) Extracted ion chromatogram of PE 36:2 acquired in negative ion mode; (C) Extracted ion chromatogram of TAG 52:3 acquired in positive ion mode; (D) Extracted ion chromatogram of FFA 18:1 acquired in negative ion mode; (E) Extracted ion chromatogram of GM3 36:1;2 acquired in negative ion mode;**](#_Toc121318003)

[**Table S1 List of identified phospholipids in human milk detected by UPLC-Q-TOF-MS**](#_Toc121318021)

[**Table S2 List of identified glycerides and free fatty acids in human milk detected by UPLC-Q-TOF-MS**](#_Toc121318022)

[**Table S3 List of identified gangliosides in human milk detected by UPLC-Q-TOF-MS**](#_Toc121318023)


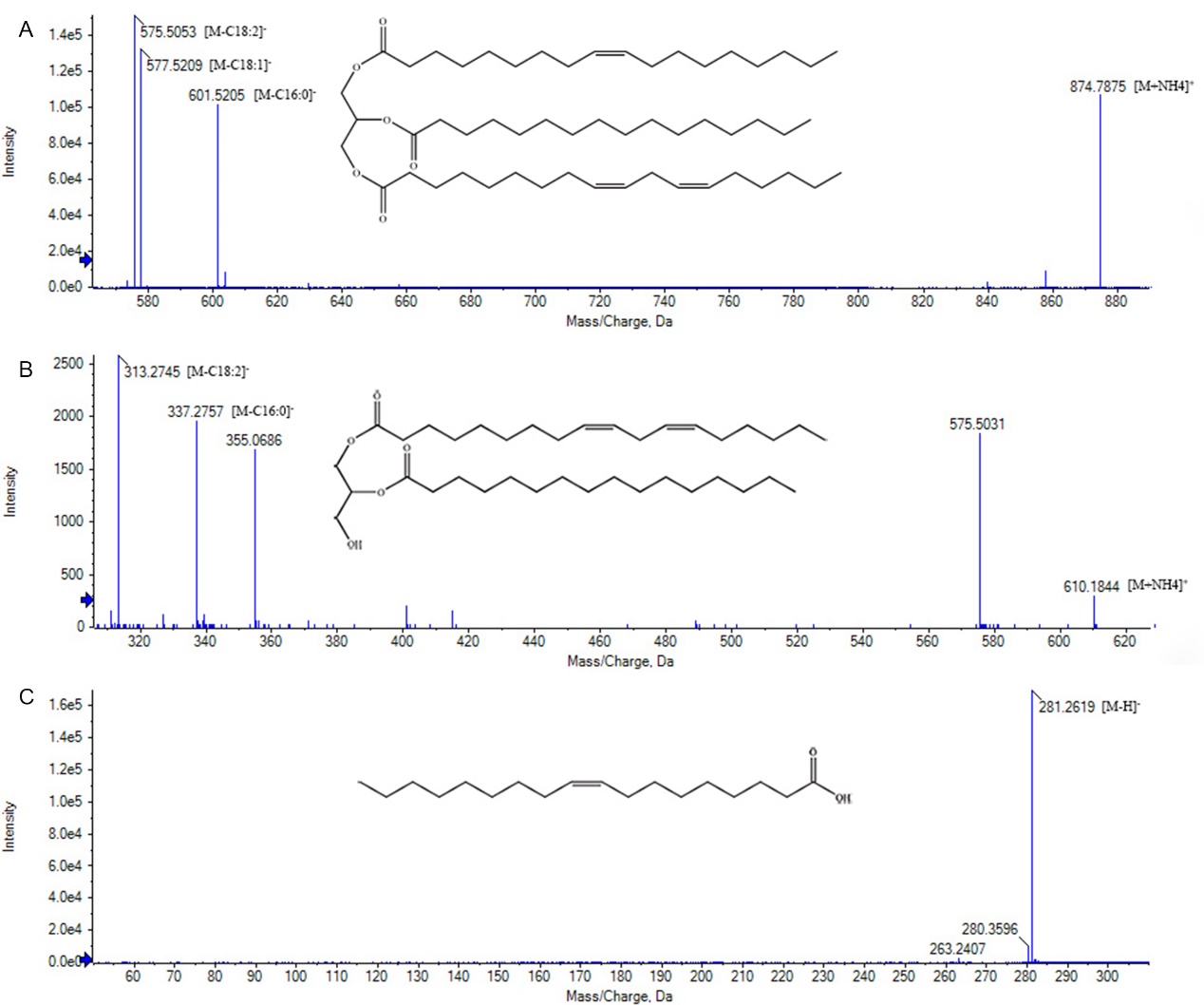


**Figure S1 (A) MS/MS fragments of TAG 52:3 acquired in positive ion mode; (B) MS/MS fragments of DAG 34:2 acquired in negative ion mode; (C) MS/MS fragments of FFA 18:1 acquired in negative ion mode.**

**
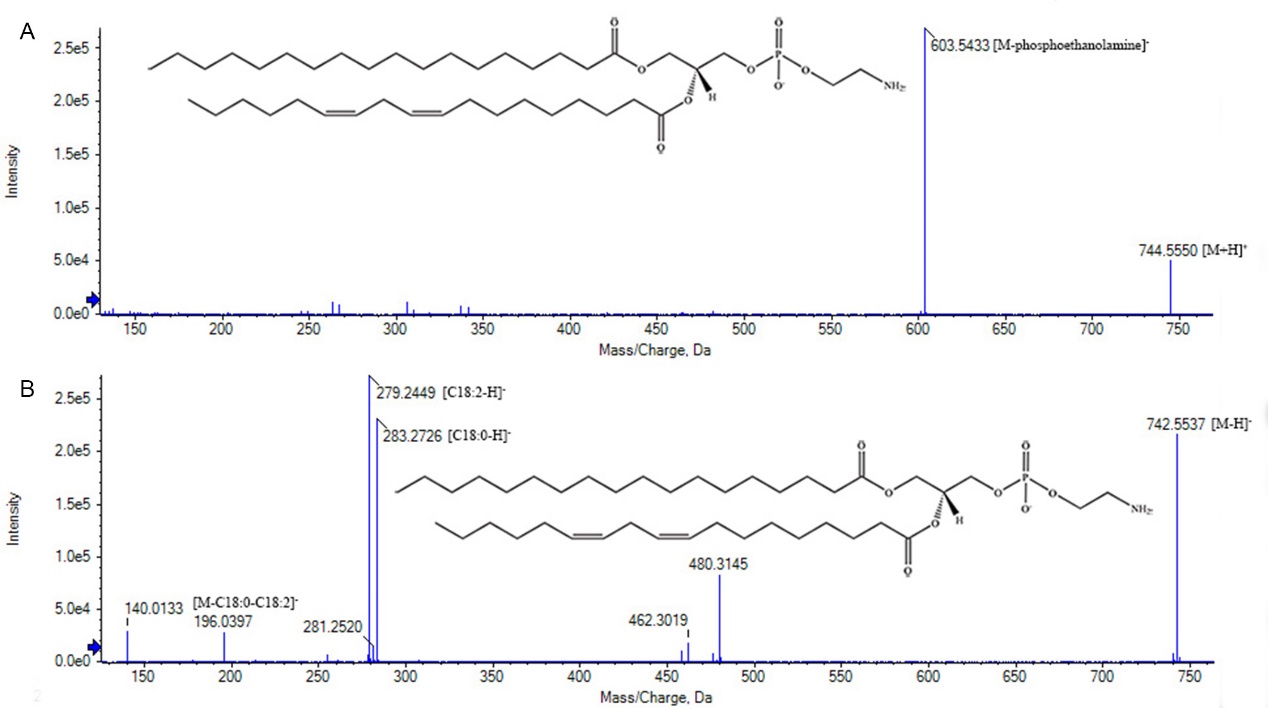
Figure S2 (A) MS/MS fragments of PE 36:2 acquired in positive ion mode; (B) MS/MS fragments of PE 36:2 acquired in negative ion mode.**


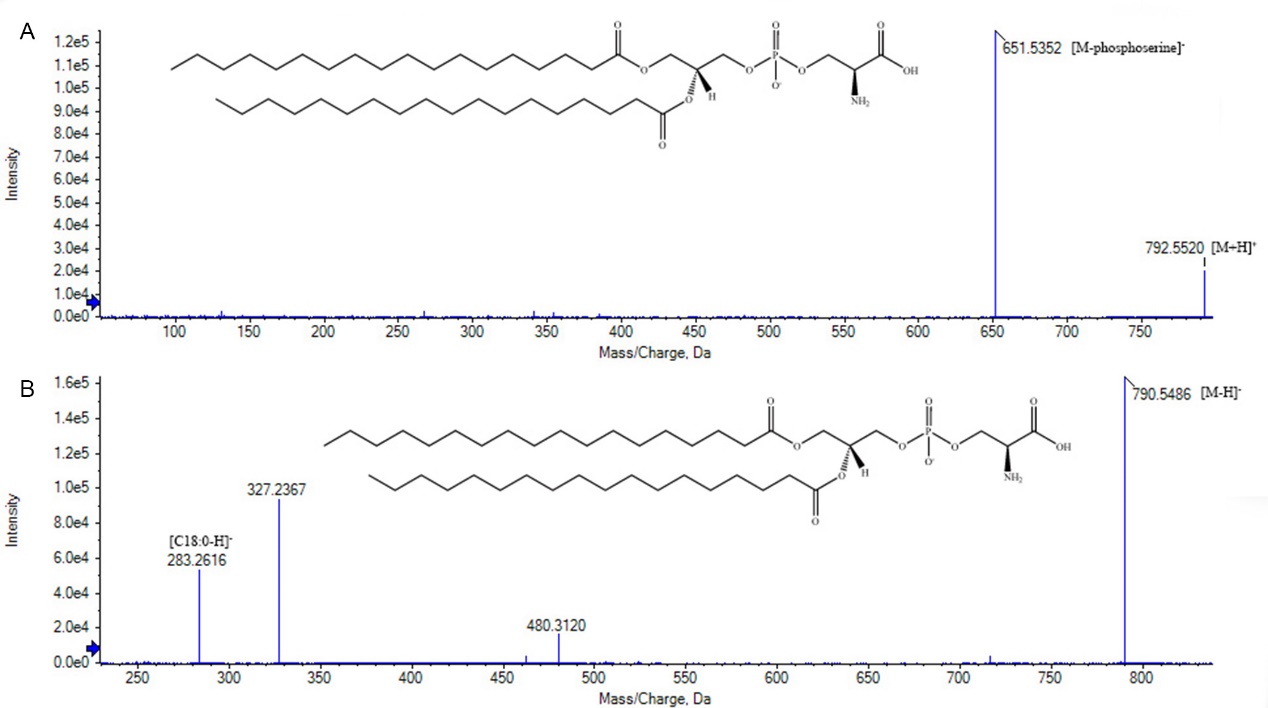


**Figure S3 (A) MS/MS fragments of PS 36:0 acquired in positive ion mode; (B) MS/MS fragments of PS 36:0 acquired in negative ion mode.**


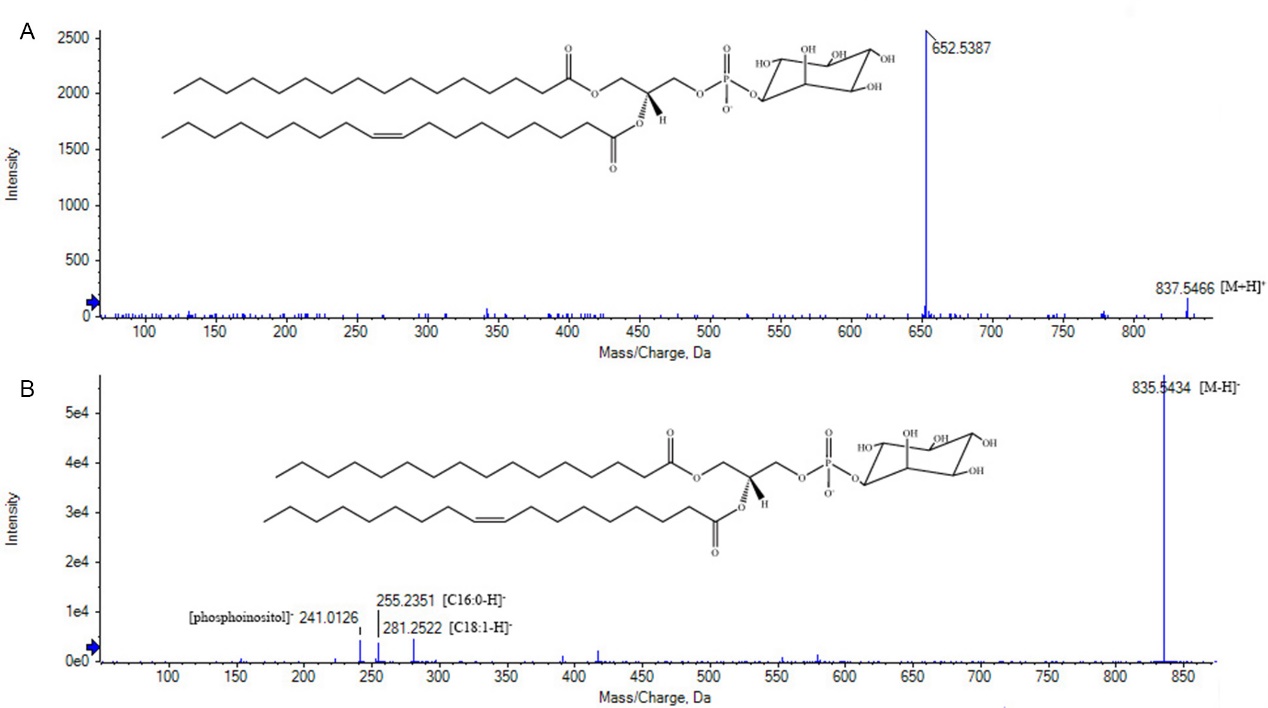


**Figure S4 (A) MS/MS fragments of PI 34:1 acquired in positive ion mode; (B) MS/MS fragments of PI 34:1 acquired in negative ion mode.**


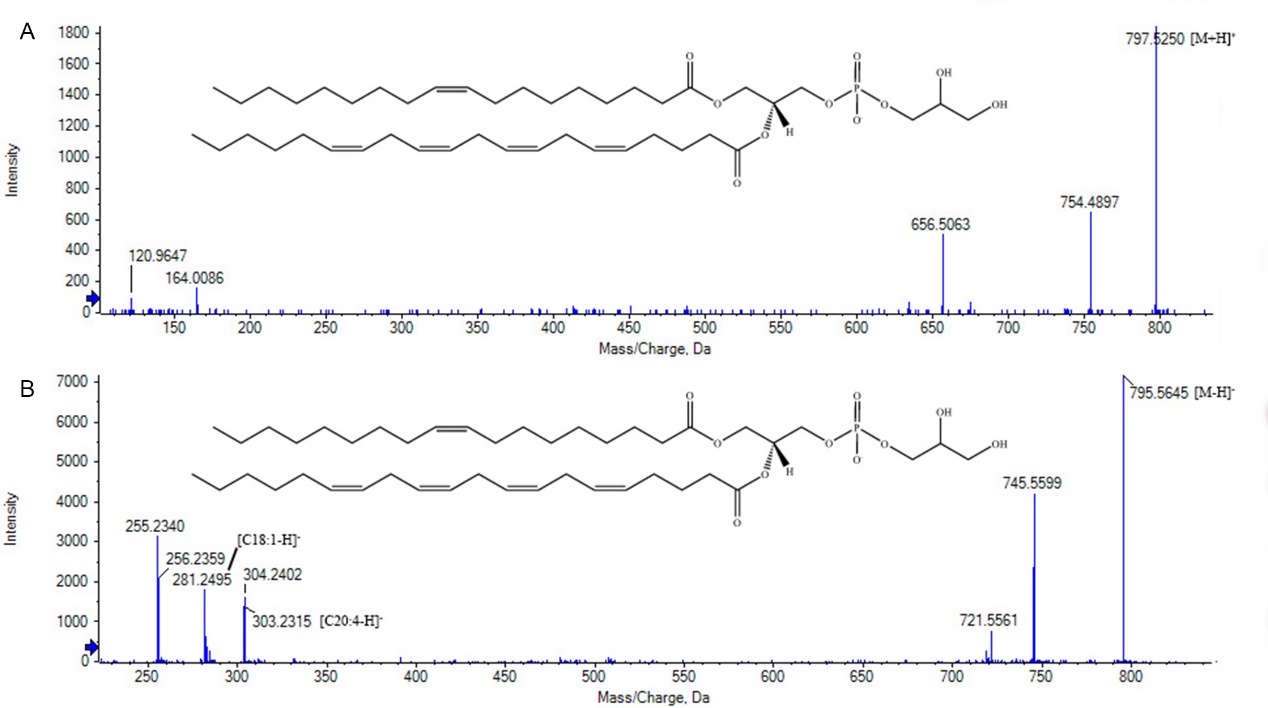


**Figure S5 (A) MS/MS fragments of PG 38:5 acquired in positive ion mode; (B) MS/MS fragments of PG 38:5 acquired in negative ion mode.**

.
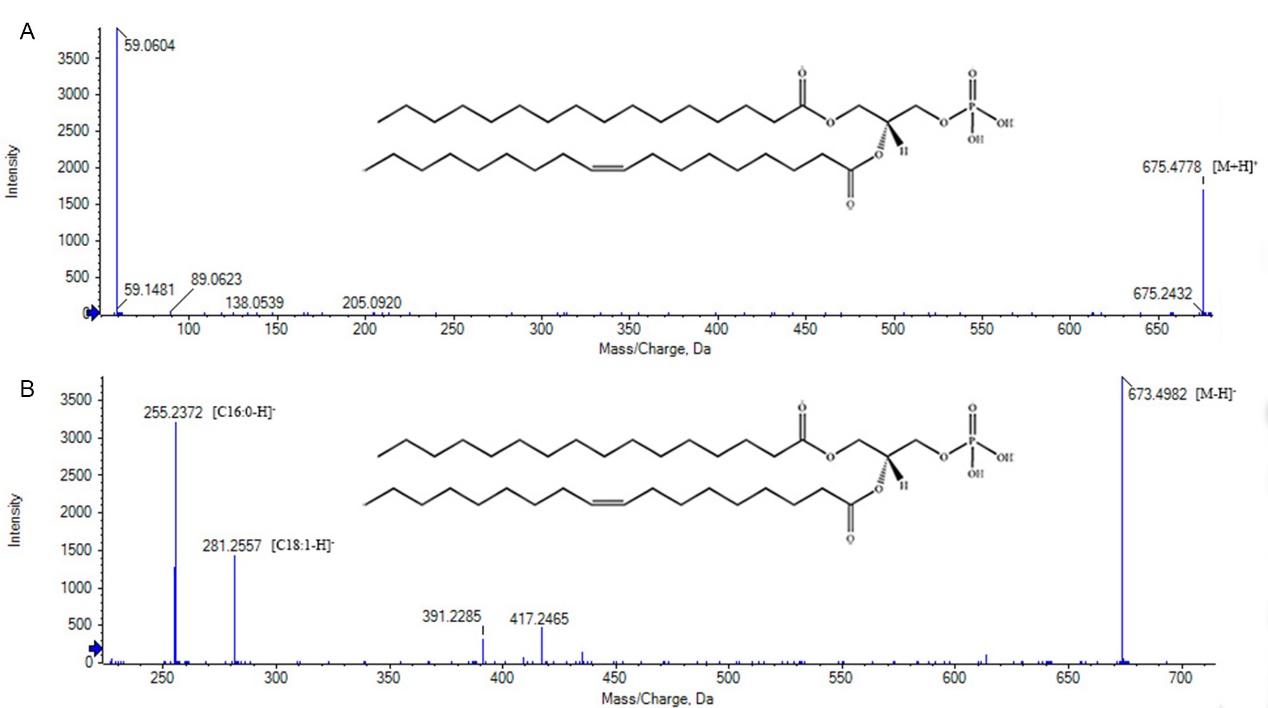


**Figure S6 (A) MS/MS fragments of PA 34:1 acquired in positive ion mode; (B) MS/MS fragments of PA 34:1 acquired in negative ion mode.**

**
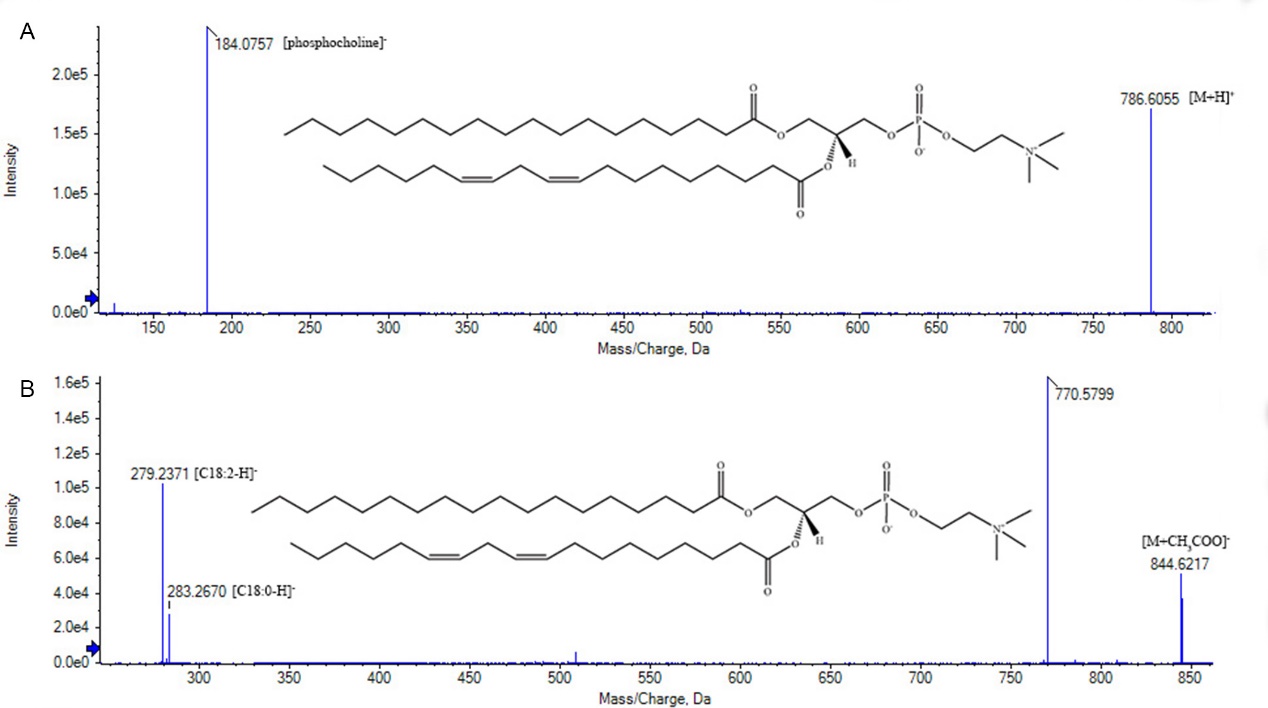
Figure S7 (A) MS/MS fragments of PC 36:2 acquired in positive ion mode; (B) MS/MS fragments of PC 36:2 acquired in negative ion mode.**


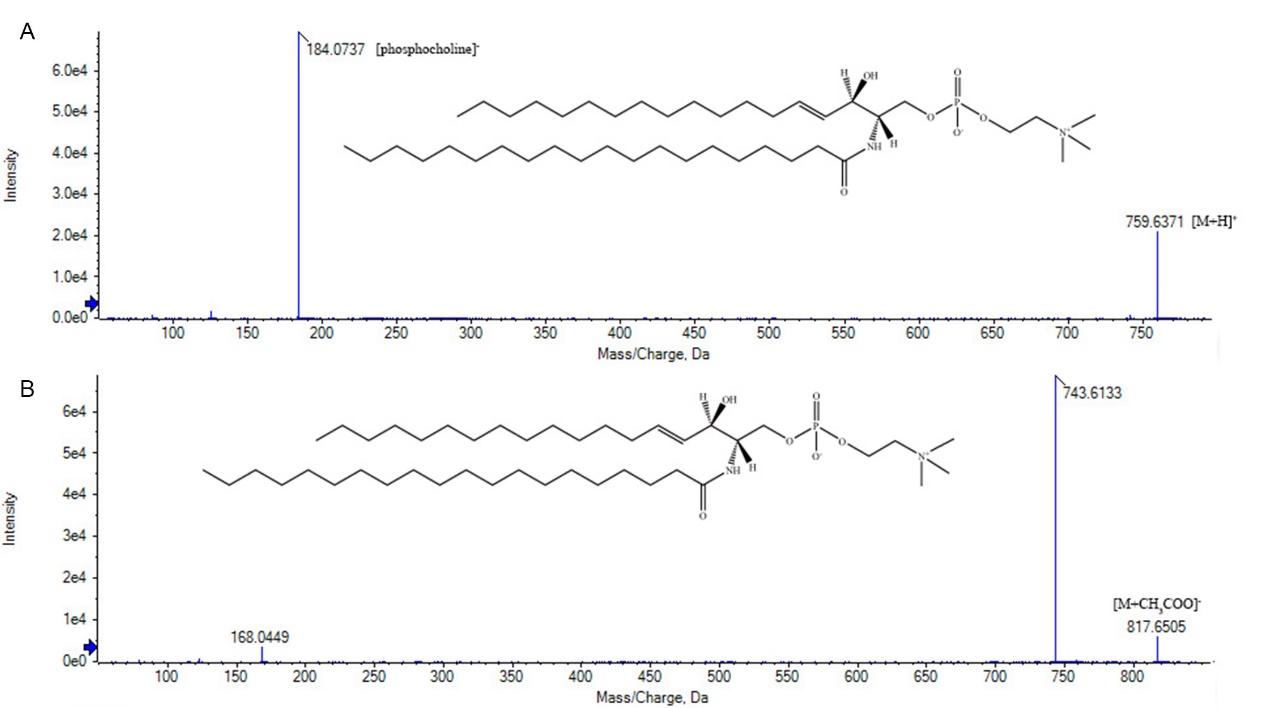


**Figure S8 (A) MS/MS fragments of SM 38:1;2 acquired in positive ion mode; (B) MS/MS fragments of SM 38:1;2 acquired in negative ion mode.**


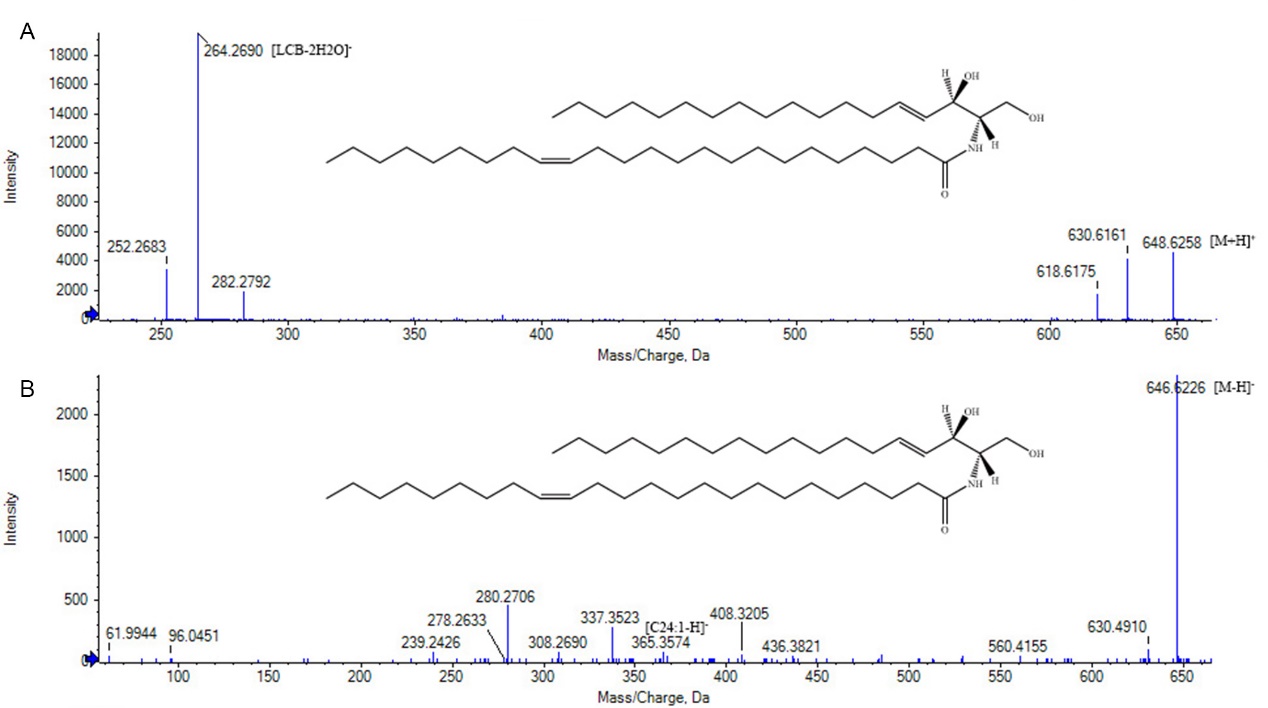


**Figure S9 (A) MS/MS fragments of Cer 42:2;2 acquired in positive ion mode; (B) MS/MS fragments of Cer 42:2;2 acquired in negative ion mode.**

**
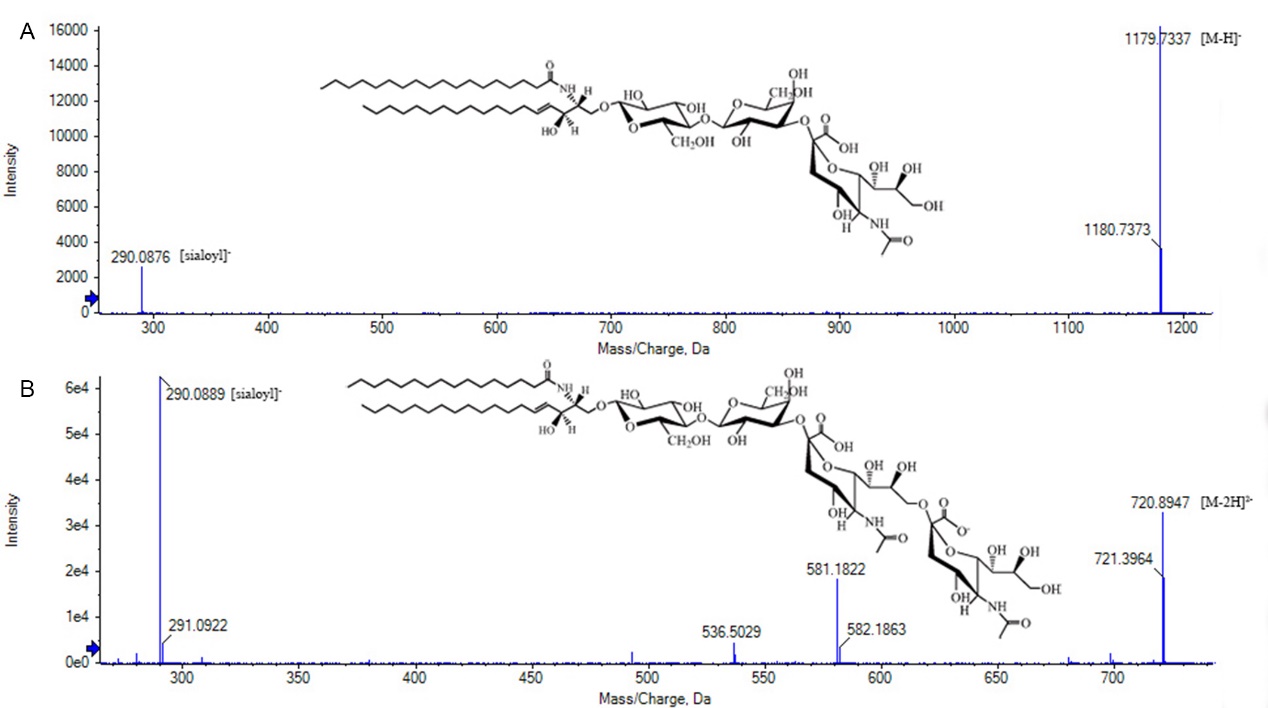
Figure S10 (A) MS/MS fragments of GM3 36:1;2 acquired in negative ion mode; (B) MS/MS fragments of GD3 34:1;2 acquired in negative ion mode.**

**
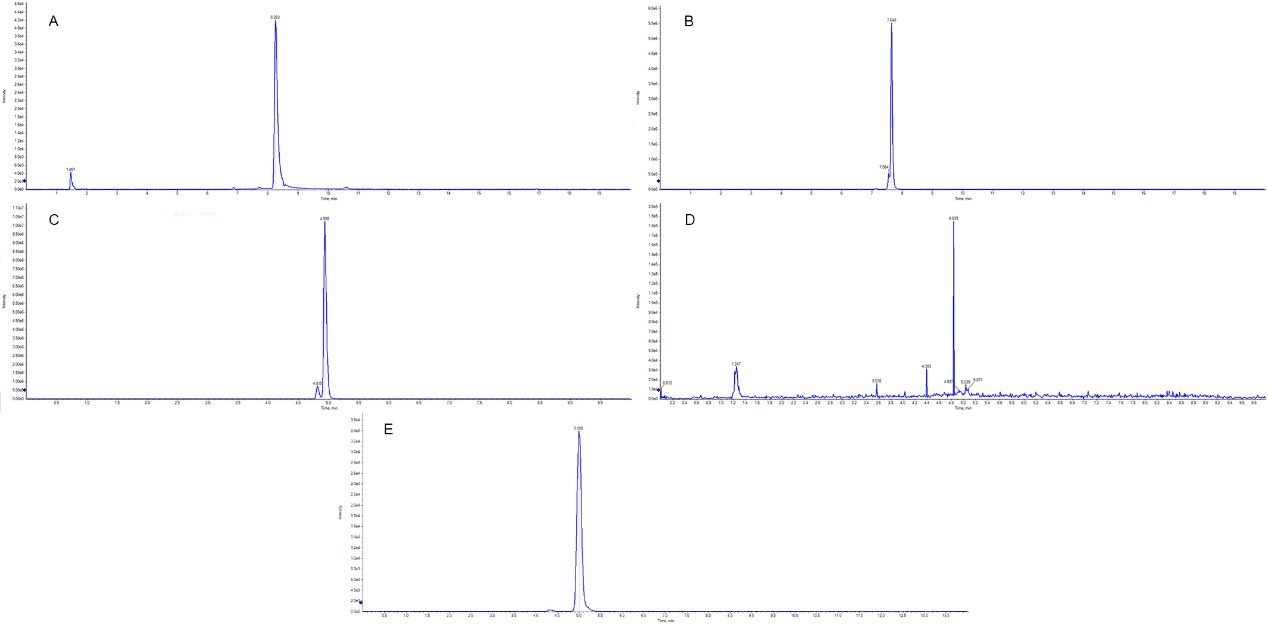
**

**Figure S11 (A) Extracted ion chromatogram of SM 38:1;2 acquired in positive ion mode; (B) Extracted ion chromatogram of PE 36:2 acquired in negative ion mode; (C) Extracted ion chromatogram of TAG 52:3 acquired in positive ion mode; (D) Extracted ion chromatogram of FFA 18:1 acquired in negative ion mode; (E) Extracted ion chromatogram of GM3 36:1;2 acquired in negative ion mode;**

**Table S1 List of identified phospholipids in human milk detected by UPLC-Q-TOF-MS**

| Lipid | Formula | m/z | Retention time | Fatty acyl |
| --- | --- | --- | --- | --- |
| PC 30:0 | C38H76NO8P | 705.5309 | 7.45 | 14:0/16:0 |
| PC 32:0 | C40H80NO8P | 733.5622 | 8.46 | 16:0/16:0 |
| PC 32:1 | C40H78NO8P | 731.5465 | 7.44 | 15:1/17:0 |
|  |  |  |  | 14:1/18:0 |
| PC 32:4 | C40H72NO8P | 725.4996 | 8.03 | 14:0/18:4 |
| PC 34:0 | C42H84NO8P | 761.5935 | 9.36 | 17:0/17:0 |
| PC 34:1 | C42H82NO8P | 759.5778 | 8.35 | 16:0/18:1 |
|  |  |  |  | 13:1/21:0 |
| PC 34:2 | C42H80NO8P | 757.5622 | 7.69 | 16:0/18:2 |
| PC 34:3 | C42H78NO8P | 755.5465 | 7.67 | 16:0/18:3 |
| PC 34:4 | C42H76NO8P | 753.5309 | 8.07 | 16:1/18:3 |
| PC 36:1 | C44H86NO8P | 787.6091 | 8.93 | 16:0/20:1 |
|  |  |  |  | 18:0/18:1 |
| PC 36:2 | C44H84NO8P | 785.5935 | 8.58 | 18:0/18:2 |
| PC 36:3 | C44H82NO8P | 783.5778 | 7.76 | 18:1/18:2 |
|  |  |  |  | 16:0/20:3 |
| PC 36:4 | C44H80NO8P | 781.5622 | 7.57 | 16:0/20:4 |
|  |  |  |  | 16:1/20:3 |
|  |  |  |  | 18:1/18:3 |
|  |  |  |  | 18:2/18:2 |
| PC 38:3 | C46H86NO8P | 811.6091 | 8.93 | 18:0/20:3 |
| PC 38:4 | C46H84NO8P | 809.5935 | 8.31 | 16:0/22:4 |
|  |  |  |  | 18:0/20:4 |
|  |  |  |  | 18:1/20:3 |
|  |  |  |  | 18:2/20:2 |
|  |  |  |  | 18:3/20:1 |
| PC 38:5 | C46H82NO8P | 807.5778 | 7.55 | 16:0/22:5 |
|  |  |  |  | 18:2/20:3 |
|  |  |  |  | 18:3/20:2 |
| PC 40:5 | C48H86NO8P | 835.6091 | 8.51 | 18:1/22:4 |
|  |  |  |  | 20:1/20:4 |
| PC 40:6 | C48H84NO8P | 833.5935 | 8.19 | 18:0/22:6 |
| LPC 18:0 | C26H54NO7P | 523.3638 | 4.08 | 18:0 |
| LPC 18:3 | C26H48NO7P | 517.3168 | 6.49 | 18:3 |
| LPC 20:2 | C28H54NO7P | 547.3638 | 8.43 | 20:2 |
| PE 26:0 | C31H62NO8P | 607.4213 | 5.40 | 12:0/14:0 |
| PE 26:1 | C31H60NO8P | 605.4057 | 4.89 | 10:0/16:1 |
| PE 28:0 | C33H66NO8P | 635.4526 | 6.10 | 12:0/16:0 |
|  |  |  |  | 14:0/14:0 |
| PE 28:1 | C33H64NO8P | 633.4370 | 5.52 | 12:0/16:1 |
| PE 28:2 | C33H62NO8P | 631.4213 | 4.93 | 10:0/18:2 |
| PE 30:0 | C35H70NO8P | 663.4839 | 6.77 | 12:0/18:0 |
|  |  |  |  | 14:0/16:0 |
|  |  |  |  | 15:0/15:0 |
| PE 30:1 | C35H68NO8P | 661.4683 | 6.21 | 12:0/18:1 |
|  |  |  |  | 14:0/16:1 |
| PE 30:4 | C35H62NO8P | 655.4213 | 2.67 | 14:0/16:4 |
| PE 32:0 | C37H74NO8P | 691.5152 | 7.41 | 16:0/16:0 |
| PE 32:1 | C37H72NO8P | 689.4996 | 6.87 | 14:0/18:1 |
|  |  |  |  | 16:0/16:1 |
| PE 32:2 | C37H70NO8P | 687.4839 | 6.37 | 14:0/18:2 |
|  |  |  |  | 16:1/16:1 |
| PE 32:3 | C37H68NO8P | 685.4683 | 5.83 | 16:1/16:2 |
| PE 32:4 | C37H66NO8P | 683.4526 | 5.60 | 16:0/16:4 |
| PE 34:0 | C39H78NO8P | 719.5465 | 7.98 | 16:0/18:0 |
| PE 34:1 | C39H76NO8P | 717.5309 | 7.48 | 16:0/18:1 |
| PE 34:2 | C39H74NO8P | 715.5152 | 7.02 | 15:1/19:1 |
|  |  |  |  | 16:0/18:2 |
|  |  |  |  | 16:1/18:1 |
| PE 34:3 | C39H72NO8P | 713.4996 | 6.60 | 16:0/18:3 |
|  |  |  |  | 16:1/18:2 |
| PE 34:4 | C39H70NO8P | 711.4839 | 6.04 | 16:2/18:2 |
|  |  |  |  | 16:1/18:3 |
| PE 36:0 | C41H82NO8P | 747.5778 | 8.06 | 16:0/20:0 |
|  |  |  |  | 18:0/18:0 |
| PE 36:1 | C41H80NO8P | 745.5622 | 8.04 | 16:0/20:1 |
|  |  |  |  | 18:0/18:1 |
| PE 36:2 | C41H78NO8P | 743.5465 | 7.63 | 17:1/19:1 |
|  |  |  |  | 18:0/18:2 |
|  |  |  |  | 18:1/18:1 |
| PE 36:3 | C41H76NO8P | 741.5309 | 7.10 | 16:0/20:3 |
|  |  |  |  | 18:0/18:3 |
|  |  |  |  | 18:1/18:2 |
| PE 36:4 | C41H74NO8P | 739.5152 | 6.83 | 16:0/20:4 |
|  |  |  |  | 18:1/18:3 |
|  |  |  |  | 18:2/18:2 |
| PE 36:5 | C41H72NO8P | 737.4996 | 6.67 | 18:2/18:3 |
| PE 36:6 | C41H70NO8P | 735.4839 | 6.10 | 14:0/22:6 |
| PE 38:0 | C43H86NO8P | 775.6091 | 8.65 | 18:0/20:0 |
| PE 38:1 | C43H84NO8P | 773.5935 | 8.54 | 18:0/20:1 |
|  |  |  |  | 18:1/20:0 |
| PE 38:2 | C43H82NO8P | 771.5778 | 8.12 | 18:0/20:2 |
|  |  |  |  | 18:1/20:1 |
|  |  |  |  | 19:1/19:1 |
|  |  |  |  | 18:2/20:0 |
| PE 38:3 | C43H80NO8P | 769.5622 | 7.79 | 18:0/20:3 |
|  |  |  |  | 18:1/20:2 |
|  |  |  |  | 18:2/20:1 |
| PE 38:4 | C43H78NO8P | 767.5465 | 7.54 | 16:0/22:4 |
|  |  |  |  | 18:0/20:4 |
|  |  |  |  | 18:1/20:3 |
|  |  |  |  | 18:2/20:2 |
| PE 38:5 | C43H76NO8P | 765.5309 | 7.01 | 16:0/22:5 |
|  |  |  |  | 18:0/20:5 |
|  |  |  |  | 18:1/20:4 |
|  |  |  |  | 18:2/20:3 |
| PE 38:6 | C43H74NO8P | 763.5152 | 6.80 | 16:0/22:6 |
|  |  |  |  | 16:1/22:5 |
|  |  |  |  | 18:2/20:4 |
| PE 40:0 | C45H90NO8P | 803.6404 | 9.44 | 18:0/22:0 |
| PE 40:1 | C45H88NO8P | 801.6248 | 9.01 | 18:0/22:1 |
|  |  |  |  | 18:1/22:0 |
| PE 40:2 | C45H86NO8P | 799.6091 | 8.61 | 18:0/22:2 |
|  |  |  |  | 18:1/22:1 |
|  |  |  |  | 20:1/20:1 |
| PE 40:3 | C45H84NO8P | 797.5935 | 8.16 | 18:1/22:2 |
|  |  |  |  | 18:2/22:1 |
| PE 40:4 | C45H82NO8P | 795.5778 | 7.98 | 18:0/22:4 |
|  |  |  |  | 20:0/20:4 |
| PE 40:5 | C45H80NO8P | 793.5622 | 7.76 | 18:0/22:5 |
|  |  |  |  | 18:1/22:4 |
|  |  |  |  | 20:1/20:4 |
| PE 40:6 | C45H78NO8P | 791.5465 | 7.41 | 18:0/22:6 |
|  |  |  |  | 18:1/22:5 |
|  |  |  |  | 18:2/22:4 |
| PE 42:1 | C47H92NO8P | 829.6561 | 9.48 | 18:0/24:1 |
|  |  |  |  | 18:1/24:0 |
| PE 42:2 | C47H90NO8P | 827.6404 | 9.06 | 18:0/24:2 |
|  |  |  |  | 18:1/24:1 |
|  |  |  |  | 20:1/22:1 |
|  |  |  |  | 18:2/24:0 |
| PE 42:3 | C47H88NO8P | 825.6248 | 8.61 | 18:1/24:2 |
|  |  |  |  | 18:2/24:1 |
| PE 42:4 | C47H86NO8P | 823.6091 | 8.41 | 18:0/24:4 |
| LPE 14:0 | C19H40NO7P | 425.2542 | 2.40 | 14:0 |
| LPE 14:1 | C19H38NO7P | 423.2386 | 2.14 | 14:1 |
| LPE 16:0 | C21H44NO7P | 453.2855 | 2.86 | 16:0 |
| LPE 16:1 | C21H42NO7P | 451.2699 | 2.46 | 16:1 |
| LPE 18:0 | C23H48NO7P | 481.3168 | 3.65 | 18:0 |
| LPE 18:1 | C23H46NO7P | 479.3012 | 2.95 | 18:1 |
| LPE 18:2 | C23H44NO7P | 477.2855 | 2.57 | 18:2 |
| LPE 20:0 | C25H52NO7P | 509.3481 | 4.40 | 20:0 |
| LPE 20:1 | C25H50NO7P | 507.3325 | 3.59 | 20:1 |
| LPE 20:2 | C25H48NO7P | 505.3168 | 3.12 | 20:2 |
| LPE 20:4 | C25H44NO7P | 501.2855 | 2.51 | 20:4 |
| LPE 22:0 | C27H56NO7P | 537.3794 | 5.29 | 22:0 |
| LPE 22:1 | C27H54NO7P | 535.3638 | 4.33 | 22:1 |
| LPE 22:2 | C27H52NO7P | 533.3481 | 3.72 | 22:2 |
| LPE 22:6 | C27H44NO7P | 525.2855 | 2.45 | 22:6 |
| LPE 24:0 | C29H60NO7P | 565.4107 | 6.07 | 24:0 |
| LPE 24:1 | C29H58NO7P | 563.3951 | 5.27 | 24:1 |
| PI 32:0 | C41H79O13P | 810.5258 | 5.55 | 14:0/18:0 |
|  |  |  |  | 16:0/16:0 |
| PI 34:0 | C43H83O13P | 838.5571 | 6.05 | 16:0/18:0 |
| PI 34:1 | C43H81O13P | 836.5415 | 5.61 | 16:0/18:1 |
|  |  |  |  | 16:1/18:0 |
| PI 34:2 | C43H79O13P | 834.5258 | 5.21 | 16:0/18:2 |
| PI 36:0 | C45H87O13P | 866.5884 | 6.40 | 18:0/18:0 |
| PI 36:1 | C45H85O13P | 864.5728 | 6.25 | 18:0/18:1 |
| PI 36:2 | C45H83O13P | 862.5571 | 5.84 | 18:0/18:2 |
| PI 36:3 | C45H81O13P | 860.5415 | 5.31 | 16:0/20:3 |
|  |  |  |  | 18:0/18:3 |
|  |  |  |  | 18:1/18:2 |
| PI 36:4 | C45H79O13P | 858.5258 | 7.41 | 16:0/20:4 |
|  |  |  |  | 18:2/18:2 |
| PI 38:2 | C47H87O13P | 890.5884 | 6.26 | 18:0/20:2 |
| PI 38:3 | C47H85O13P | 888.5728 | 5.97 | 18:0/20:3 |
| PI 38:4 | C47H83O13P | 886.5571 | 5.78 | 16:0/22:4 |
|  |  |  |  | 18:0/20:4 |
|  |  |  |  | 18:1/20:3 |
| PI 38:5 | C47H81O13P | 884.5415 | 5.22 | 18:0/20:5 |
|  |  |  |  | 18:1/20:4 |
|  |  |  |  | 18:2/20:3 |
| PI 40:5 | C49H85O13P | 912.5728 | 5.78 | 18:0/22:5 |
| PI 40:6 | C49H83O13P | 910.5571 | 5.49 | 18:0/22:6 |
|  |  |  |  | 20:2/20:4 |
|  |  |  |  | 20:3/20:3 |
| LPI 16:0 | C25H49O12P | 572.2962 | 2.06 | 16:0 |
| LPI 18:0 | C27H53O12P | 600.3275 | 2.45 | 18:0 |
| LPI 20:2 | C29H53O12P | 624.3275 | 2.30 | 20:2 |
| PS 32:0 | C38H74NO10P | 735.5050 | 6.87 | 16:0/16:0 |
| PS 32:2 | C38H70NO10P | 731.4737 | 7.28 | 16:1/16:1 |
|  |  |  |  | 12:2/20:0 |
| PS 34:0 | C40H78NO10P | 763.5363 | 6.77 | 16:0/18:0 |
| PS 34:1 | C40H76NO10P | 761.5207 | 6.61 | 16:1/18:0 |
| PS 34:2 | C40H74NO10P | 759.5050 | 7.85 | 16:0/18:2 |
| PS 36:0 | C42H82NO10P | 791.5676 | 7.43 | 18:0/18:0 |
| PS 36:1 | C42H80NO10P | 789.5520 | 6.93 | 18:0/18:1 |
| PS 36:2 | C42H78NO10P | 787.5363 | 6.47 | 18:0/18:2 |
|  |  |  |  | 18:1/18:1 |
| PS 36:3 | C42H76NO10P | 785.5207 | 8.00 | 18:0/18:3 |
|  |  |  |  | 18:1/18:2 |
| PS 36:4 | C42H74NO10P | 783.5050 | 7.49 | 18:0/18:4 |
| PS 38:2 | C44H82NO10P | 815.5676 | 8.38 | 18:0/20:2 |
|  |  |  |  | 18:1/20:1 |
|  |  |  |  | 18:2/20:0 |
| PS 38:3 | C44H80NO10P | 813.5520 | 6.53 | 18:0/20:3 |
|  |  |  |  | 17:2/21:1 |
| PS 38:5 | C44H76NO10P | 809.5207 | 7.93 | 18:0/20:5 |
|  |  |  |  | 18:1/20:4 |
|  |  |  |  | 18:2/20:3 |
| PS 38:6 | C44H74NO10P | 807.5050 | 7.37 | 18:2/20:4 |
| PS 40:3 | C46H84NO10P | 841.5833 | 7.66 | 18:0/22:3 |
|  |  |  |  | 18:2/22:1 |
| PS 40:4 | C46H82NO10P | 839.5676 | 6.77 | 18:0/22:4 |
| PS 40:6 | C46H78NO10P | 835.5363 | 6.13 | 18:0/22:6 |
| PS 44:2 | C50H94NO10P | 899.6615 | 9.79 | 22:1/22:1 |
| LPS 18:0 | C24H48NO9P | 525.3067 | 2.81 | 18:0 |
| LPS 22:1 | C28H54NO9P | 579.3536 | 6.52 | 22:1 |
| LPS 22:2 | C28H52NO9P | 577.3380 | 6.46 | 22:2 |
| PG 30:0 | C36H71O10P | 694.4785 | 4.97 | 14:0/16:0 |
|  |  |  |  | 15:0/15:0 |
| PG 32:0 | C38H75O10P | 722.5098 | 6.27 | 16:0/16:0 |
|  |  |  |  | 14:0/18:0 |
| PG 32:1 | C38H73O10P | 720.4941 | 5.16 | 14:0/18:1 |
|  |  |  |  | 16:0/16:1 |
| PG 32:2 | C38H71O10P | 718.4785 | 4.55 | 14:0/18:2 |
|  |  |  |  | 16:1/16:1 |
| PG 34:0 | C40H79O10P | 750.5411 | 7.33 | 16:0/18:0 |
| PG 34:1 | C40H77O10P | 748.5254 | 6.40 | 16:0/18:1 |
| PG 34:2 | C40H75O10P | 746.5098 | 5.19 | 16:0/18:2 |
|  |  |  |  | 16:1/18:1 |
| PG 36:1 | C42H81O10P | 776.5567 | 7.74 | 18:0/18:1 |
| PG 36:2 | C42H79O10P | 774.5411 | 6.00 | 17:1/19:1 |
|  |  |  |  | 18:0/18:2 |
|  |  |  |  | 18:1/18:1 |
| PG 36:3 | C42H77O10P | 772.5254 | 5.10 | 18:0/18:3 |
|  |  |  |  | 18:1/18:2 |
| PG 36:4 | C42H75O10P | 770.5098 | 5.84 | 18:2/18:2 |
| PG 38:1 | C44H85O10P | 804.5880 | 7.48 | 18:0/20:1 |
| PG 38:2 | C44H83O10P | 802.5724 | 7.96 | 18:0/20:2 |
| PG 38:4 | C44H79O10P | 798.5411 | 5.98 | 16:0/22:4 |
|  |  |  |  | 18:0/20:4 |
| PG 38:5 | C44H77O10P | 796.5254 | 8.38 | 18:1/20:4 |
| PG 40:6 | C46H79O10P | 822.5411 | 5.73 | 18:0/22:6 |
| PG 42:3 | C48H89O10P | 856.6193 | 8.55 | 20:1/22:2 |
| PG 44:4 | C50H91O10P | 882.6350 | 8.23 | 22:2/22:2 |
| LPG 16:0 | C22H45O9P | 484.2801 | 2.09 | 16:0 |
| LPG 18:0 | C24H49O9P | 512.3114 | 2.59 | 18:0 |
| PA 28:0 | C31H61NO8P | 592.4104 | 7.35 | 12:0/16:0 |
| PA 32:0 | C35H69NO8P | 648.4730 | 8.33 | 16:0/16:0 |
| PA 32:1 | C35H67NO8P | 646.4574 | 7.44 | 14:0/18:1 |
| PA 34:1 | C37H71NO8P | 674.4887 | 8.35 | 16:0/18:1 |
| PA 34:2 | C37H69NO8P | 672.4730 | 7.70 | 16:0/18:2 |
| PA 36:0 | C39H77NO8P | 704.5356 | 8.31 | 18:0/18:0 |
| PA 36:1 | C39H75NO8P | 702.5200 | 7.86 | 18:0/18:1 |
| PA 36:2 | C39H73NO8P | 700.5043 | 7.44 | 18:0/18:2 |
|  |  |  |  | 18:1/18:1 |
| PA 36:3 | C39H71NO8P | 698.4887 | 7.71 | 18:1/18:2 |
| PA 36:4 | C39H69NO8P | 696.4730 | 7.72 | 18:1/18:3 |
| PA 36:5 | C39H67NO8P | 694.4574 | 7.77 | 18:2/18:3 |
| PA 38:0 | C41H81NO8P | 732.5669 | 8.81 | 18:0/20:0 |
| PA 38:1 | C41H79NO8P | 730.5513 | 8.37 | 18:0/20:1 |
| PA 40:0 | C43H85NO8P | 760.5982 | 9.34 | 18:0/22:0 |
|  |  |  |  | 20:0/20:0 |
| PA 40:1 | C43H83NO8P | 758.5826 | 8.86 | 20:0/20:1 |
|  |  |  |  | 18:0/22:1 |
| PA 40:2 | C43H81NO8P | 756.5669 | 8.54 | 20:1/20:1 |
|  |  |  |  | 18:0/22:2 |
|  |  |  |  | 20:0/20:2 |
| PA 40:3 | C43H79NO8P | 754.5513 | 8.15 | 20:1/20:2 |
| PA 40:5 | C43H75NO8P | 750.5200 | 7.33 | 18:0/22:5 |
| PA 40:6 | C43H73NO8P | 748.5043 | 7.21 | 18:0/22:6 |
| PA 42:2 | C45H85NO8P | 784.5982 | 8.99 | 20:1/22:1 |
| PA 42:3 | C45H83NO8P | 782.5826 | 8.59 | 20:1/22:2 |
| PA 44:2 | C47H89NO8P | 812.6295 | 9.42 | 22:1/22:1 |
| LPA 16:0 | C19H39NO7P | 410.2433 | 5.10 | 16:0 |
| LPA 18:0 | C21H43NO7P | 438.2746 | 3.03 | 18:0 |
| SM 28:0:2 | C33H69N2O6P | 620.4893 | 5.11 | d18:0/10:0 |
| SM 30:0:2 | C35H73N2O6P | 648.5206 | 5.91 | d18:0/12:0 |
| SM 30:1:2 | C35H71N2O6P | 646.5050 | 5.58 | d18:1/12:1 |
| SM 30:2:2 | C35H69N2O6P | 644.4893 | 5.89 | d18:1/12:0 |
| SM 32:0:2 | C37H77N2O6P | 676.5519 | 6.72 | d18:0/14:0 |
| SM 32:0:3 | C37H77N2O7P | 692.5468 | 8.47 | t18:0/14:0 |
| SM 32:1:2 | C37H75N2O6P | 674.5363 | 6.38 | d18:1/14:0 |
| SM 32:1:3 | C37H75N2O7P | 690.5312 | 8.11 | t18:1/14:0 |
| SM 34:0:2 | C39H81N2O6P | 704.5832 | 7.56 | d18:0/16:0 |
| SM 34:0:3 | C39H81N2O7P | 720.5781 | 9.50 | t18:0/16:0 |
| SM 34:1:2 | C39H79N2O6P | 702.5676 | 7.20 | d18:1/16:0 |
| SM 34:1:3 | C39H79N2O7P | 718.5625 | 7.24 | t18:1/16:0 |
| SM 34:2:2 | C39H77N2O6P | 700.5519 | 6.84 | d18:1/16:1 |
| SM 34:2:3 | C39H77N2O7P | 716.5468 | 7.63 | t18:1/16:1 |
| SM 36:0:2 | C41H85N2O6P | 732.6145 | 8.31 | d18:0/18:0 |
| SM 36:0:3 | C41H85N2O7P | 748.6094 | 9.60 | t18:0/18:0 |
| SM 36:1:2 | C41H83N2O6P | 730.5989 | 8.05 | d18:0/18:1 |
|  |  |  |  | d18:1/18:0 |
| SM 36:1:3 | C41H83N2O7P | 746.5938 | 9.48 | t18:0/18:1 |
|  |  |  |  | t18:1/18:0 |
| SM 36:2:2 | C41H81N2O6P | 728.5832 | 7.31 | d18:1/18:1 |
| SM 36:2:3 | C41H81N2O7P | 744.5781 | 8.53 | t18:1/18:1 |
| SM 38:0:2 | C43H89N2O6P | 760.6458 | 9.10 | d18:0/20:0 |
| SM 38:0:3 | C43H89N2O7P | 776.6407 | 9.55 | t18:0/20:0 |
| SM 38:1:2 | C43H87N2O6P | 758.6302 | 8.94 | d18:0/20:1 |
|  |  |  |  | d18:1/20:0 |
| SM 38:1:3 | C43H87N2O7P | 774.6251 | 9.81 | t18:0/20:1 |
|  |  |  |  | t18:1/20:0 |
| SM 38:2:2 | C43H85N2O6P | 756.6145 | 8.15 | d18:0/20:2 |
|  |  |  |  | d18:1/20:1 |
| SM 38:2:3 | C43H85N2O7P | 772.6094 | 9.52 | t18:0/20:2 |
|  |  |  |  | t18:1/20:1 |
| SM 40:0:2 | C45H93N2O6P | 788.6771 | 10.10 | d18:0/22:0 |
| SM 40:0:3 | C45H93N2O7P | 804.6720 | 10.13 | t18:0/22:0 |
| SM 40:1:2 | C45H91N2O6P | 786.6615 | 9.97 | d18:1/22:0 |
| SM 40:1:3 | C45H91N2O7P | 802.6564 | 8.82 | t18:1/22:0 |
| SM 40:2:2 | C45H89N2O6P | 784.6458 | 9.01 | d18:0/22:2 |
|  |  |  |  | d18:1/22:1 |
| SM 40:2:3 | C45H89N2O7P | 800.6407 | 9.74 | t18:0/22:2 |
|  |  |  |  | t18:1/22:1 |
| SM 42:0:2 | C47H97N2O6P | 816.7084 | 10.26 | d18:0/24:0 |
| SM 42:0:3 | C47H97N2O7P | 832.7033 | 10.45 | t18:0/24:0 |
| SM 42:1:2 | C47H95N2O6P | 814.6928 | 11.59 | d18:0/24:1 |
|  |  |  |  | d18:1/24:0 |
| SM 42:1:3 | C47H95N2O7P | 830.6877 | 9.72 | t18:0/24:1 |
|  |  |  |  | t18:1/24:0 |
| SM 42:2:2 | C47H93N2O6P | 812.6771 | 9.89 | d18:0/24:2 |
|  |  |  |  | d18:1/24:1 |
| SM 42:2:3 | C47H93N2O7P | 828.6720 | 8.31 | t18:0/24:2 |
|  |  |  |  | t18:1/24:1 |
| SM 44:0:2 | C49H101N2O6P | 844.7397 | 10.52 | d18:0/26:0 |
| SM 44:0:3 | C49H101N2O7P | 860.7346 | 10.90 | t18:0/26:0 |
| SM 44:1:2 | C49H99N2O6P | 842.7241 | 8.38 | d18:0/26:1 |
|  |  |  |  | d18:1/26:0 |
| SM 44:1:3 | C49H99N2O7P | 858.7190 | 9.08 | t18:0/26:1 |
|  |  |  |  | t18:1/26:0 |
| SM 44:2:2 | C49H97N2O6P | 840.7084 | 11.11 | d18:1/26:1 |
| SM 44:2:3 | C49H97N2O7P | 856.7033 | 10.24 | t18:1/26:1 |
| SM 46:0:2 | C51H105N2O6P | 872.7710 | 10.87 | d18:0/28:0 |
| SM 46:2:2 | C51H101N2O6P | 868.7397 | 8.39 | d18:1/28:1 |
| Cer 32:1:2 | C32H63NO3P | 509.4808 | 6.78 | d18:1/14:0 |
| Cer 34:0:2 | C34H69NO3P | 539.5278 | 6.83 | d18:0/16:0 |
| Cer 34:0:3 | C34H69NO4P | 555.5227 | 6.77 | t18:0/16:1 |
| Cer 34:1:2 | C34H67NO3P | 537.5121 | 6.79 | d18:1/16:0 |
| Cer 34:1:3 | C34H67NO4P | 553.5070 | 6.32 | t18:1/16:0 |
| Cer 34:2:2 | C34H65NO3P | 535.4965 | 6.56 | d18:1/16:1 |
| Cer 36:0:3 | C36H73NO4P | 583.5540 | 7.13 | t18:0/18:0 |
| Cer 36:1:2 | C36H71NO3P | 565.5434 | 7.36 | d18:0/18:1 |
| Cer 36:1:3 | C36H71NO4P | 581.5383 | 6.73 | t18:0/18:1 |
| Cer 36:2:2 | C36H69NO3P | 563.5278 | 7.17 | d18:1/18:1 |
| Cer 38:0:2 | C38H77NO3P | 595.5904 | 7.91 | d18:0/20:0 |
| Cer 38:0:3 | C38H77NO4P | 611.5853 | 9.01 | t18:0/20:0 |
| Cer 38:1:2 | C_38_H_75_NO_3_P | 593.5747 | 7.97 | d18:0/20:1 |
|  |  |  |  | d18:1/20:0 |
| Cer 38:1:3 | C_38_H_75_NO_4_P | 609.5696 | 9.51 | t18:0/20:1 |
|  |  |  |  | t18:1/20:0 |
| Cer 38:2:2 | C_38_H_73_NO_3_P | 591.5591 | 7.74 | d18:1/20:1 |
| Cer 40:0:2 | C_40_H_81_NO_3_P | 623.6217 | 8.42 | d18:0/22:0 |
| Cer 40:0:3 | C_40_H_81_NO_4_P | 639.6166 | 6.13 | t18:0/22:0 |
| Cer 40:1:2 | C_40_H_79_NO_3_P | 621.6060 | 6.46 | d18:0/22:1 |
|  |  |  |  | d18:1/22:0 |
| Cer 40:1:3 | C_40_H_79_NO_4_P | 637.6009 | 7.80 | t18:0/22:1 |
|  |  |  |  | t18:1/22:0 |
| Cer 40:2:2 | C_40_H_77_NO_3_P | 619.5904 | 8.23 | d18:0/22:1 |
| Cer 40:2:3 | C_40_H_77_NO_4_P | 635.5853 | 7.30 | t18:0/22:1 |
| Cer 42:0:2 | C_42_H_85_NO_3_P | 651.6530 | 8.87 | d18:0/24:0 |
| Cer 42:0:3 | C_42_H_85_NO_4_P | 667.6479 | 7.75 | t18:0/24:0 |
| Cer 42:1:2 | C_42_H_83_NO_3_P | 649.6373 | 8.50 | d18:0/24:1 |
|  |  |  |  | d18:1/24:0 |
| Cer 42:1:3 | C_42_H_83_NO_4_P | 665.6322 | 8.03 | t18:0/24:1 |
|  |  |  |  | t18:1/24:0 |
| Cer 42:2:2 | C_42_H_81_NO_3_P | 647.6217 | 8.69 | d18:1/24:1 |
| Cer 42:2:3 | C_42_H_81_NO_4_P | 663.6166 | 7.93 | t18:0/24:2 |
|  |  |  |  | t18:1/24:1 |
| Cer 44:0:2 | C_44_H_89_NO_3_P | 679.6843 | 9.62 | d18:0/26:0 |
| Cer 44:0:3 | C_44_H_89_NO_4_P | 695.6792 | 9.55 | t18:0/26:0 |
| Cer 44:1:2 | C_44_H_87_NO_3_P | 677.6686 | 8.76 | d18:0/26:1 |
|  |  |  |  | d18:1/26:0 |
| Cer 44:1:3 | C_44_H_87_NO_4_P | 693.6635 | 8.90 | t18:1/26:0 |
| Cer 44:2:2 | C_44_H_85_NO_3_P | 675.6530 | 9.13 | d18:1/26:1 |
| Cer 44:2:3 | C_44_H_85_NO_4_P | 691.6479 | 8.84 | t18:0/26:2 |
| Cer 46:0:2 | C_46_H_93_NO_3_P | 707.7156 | 9.43 | d20:0/26:0 |
| Cer 46:1:2 | C_46_H_91_NO_3_P | 705.6999 | 9.02 | d20:1/26:0 |

The symbol “/” denotes only the fatty acid composition of lipid species, and not their sn-1 and sn-2 positions on the phospholipid-backbone.

The “d” and “t” denotes the number of hydroxyl groups link on gangliosides, “d” refers to two hydroxyl groups and the “t” refers to three hydroxyl groups.

**Table S2 List of identified glycerides and free fatty acids in human milk detected by UPLC-Q-TOF-MS**

| Lipid | Formula | m/z | Retention time | Fatty acyl |
| --- | --- | --- | --- | --- |
| DAG 24:0 | C_27_H_52_O_5_ | 456.38148 | 2.1189165 | 10:0/14:0 |
|  |  |  |  | 12:0/12:0 |
| DAG 26:0 | C_29_H_56_O_5_ | 484.41278 | 2.4631665 | 10:0/16:0 |
|  |  |  |  | 12:0/14:0 |
| DAG 28:0 | C_31_H_60_O_5_ | 512.44408 | 2.804211 | 12:0/16:0 |
|  |  |  |  | 14:0/14:0 |
| DAG 28:1 | C_31_H_58_O_5_ | 510.42843 | 2.4975415 | 10:0/18:1 |
|  |  |  |  | 12:0/16:1 |
| DAG 28:2 | C_31_H_56_O_5_ | 508.41278 | 2.217525 | 10:0/18:2 |
| DAG 30:0 | C_33_H_64_O_5_ | 540.47538 | 3.1456833 | 14:0/16:0 |
| DAG 30:1 | C_33_H_62_O_5_ | 538.45973 | 2.8655057 | 12:0/18:1 |
| DAG 30:2 | C_33_H_60_O_5_ | 536.44408 | 2.6081458 | 12:0/18:2 |
| DAG 32:0 | C_35_H_68_O_5_ | 568.50668 | 3.4458333 | 14:0/18:0 |
|  |  |  |  | 16:0/16:0 |
| DAG 32:1 | C_35_H_66_O_5_ | 566.49103 | 3.1979 | 14:0/18:1 |
|  |  |  |  | 16:0/16:1 |
| DAG 32:2 | C_35_H_64_O_5_ | 564.47538 | 2.9542083 | 14:0/18:2 |
| DAG 34:0 | C_37_H_72_O_5_ | 596.53798 | 3.7149083 | 16:0/18:0 |
| DAG 34:1 | C_37_H_70_O_5_ | 594.52233 | 3.4924638 | 16:0/18:1 |
| DAG 34:2 | C_37_H_68_O_5_ | 592.50668 | 3.2725564 | 16:0/18:2 |
| DAG 34:3 | C_37_H_66_O_5_ | 590.49103 | 3.0411553 | 16:0/18:3 |
|  |  |  |  | 16:1/18:2 |
| DAG 36:0 | C_39_H_76_O_5_ | 624.56928 | 3.8489667 | 18:0/18:0 |
| DAG 36:1 | C_39_H_74_O_5_ | 622.55363 | 3.745275 | 18:0/18:1 |
| DAG 36:2 | C_39_H_72_O_5_ | 620.53798 | 3.5446833 | 18:0/18:2 |
|  |  |  |  | 18:1/18:1 |
| DAG 36:3 | C_39_H_70_O_5_ | 618.52233 | 3.3290862 | 18:1/18:2 |
| DAG 36:4 | C_39_H_68_O_5_ | 616.50668 | 3.1002665 | 18:2/18:2 |
| DAG 36:5 | C_39_H_66_O_5_ | 614.49103 | 2.838692 | 18:2/18:3 |
| DAG 38:2 | C_41_H_76_O_5_ | 648.56928 | 3.806183 | 18:2/20:0 |
| DAG 38:3 | C_41_H_74_O_5_ | 646.55363 | 3.562975 | 18:1/20:2 |
| DAG 38:4 | C_41_H_72_O_5_ | 644.53798 | 3.406333 | 18:1/20:3 |
| DAG 38:5 | C_41_H_70_O_5_ | 642.52233 | 3.29 | 18:1/20:4 |
| DAG 40:2 | C_43_H_80_O_5_ | 676.60058 | 3.9386 | 18:1/22:1 |
| DAG 40:3 | C_43_H_78_O_5_ | 674.58493 | 3.830333 | 18:2/22:1 |
| DAG 40:7 | C_43_H_70_O_5_ | 666.52233 | 3.1208613 | 18:1/22:6 |
| DAG 40:8 | C_43_H_68_O_5_ | 664.50668 | 2.8984915 | 18:2/22:6 |
| TAG 36:0 | C_39_H_74_O_6_ | 638.54854 | 3.9041557 | 12:0/12:0/12:0 |
| TAG 36:1 | C_39_H_72_O_6_ | 636.53289 | 3.7269335 | 12:0/12:0/12:1 |
| TAG 38:0 | C_41_H_78_O_6_ | 666.57984 | 4.1422 | 12:0/12:0/14:0 |
| TAG 38:1 | C_41_H_76_O_6_ | 664.56419 | 3.9612 | 12:0/12:0/14:1 |
|  |  |  |  | 12:0/12:1/14:0 |
| TAG 38:2 | C_41_H_74_O_6_ | 662.54854 | 3.7777665 | 12:0/12:0/14:2 |
|  |  |  |  | 12:0/12:1/14:1 |
|  |  |  |  | 12:0/12:2/14:0 |
|  |  |  |  | 12:1/12:1/14:0 |
| TAG 39:0 | C_42_H_80_O_6_ | 680.5955 | 4.1564277 | 12:0/12:0/15:0 |
|  |  |  |  | 12:0/13:0/14:0 |
| TAG 40:0 | C_43_H_82_O_6_ | 694.61114 | 4.354986 | 12:0/12:0/16:0 |
|  |  |  |  | 12:0/14:0/14:0 |
| TAG 40:1 | C_43_H_80_O_6_ | 692.59549 | 4.1785583 | 12:0/12:0/16:1 |
|  |  |  |  | 12:0/12:1/16:0 |
|  |  |  |  | 12:0/14:0/14:1 |
| TAG 40:2 | C_43_H_78_O_6_ | 690.57984 | 4.0103168 | 12:0/12:0/16:2 |
|  |  |  |  | 12:0/12:1/16:1 |
|  |  |  |  | 12:0/12:2/16:0 |
|  |  |  |  | 12:0/14:0/14:2 |
|  |  |  |  | 12:1/12:1/16:0 |
|  |  |  |  | 13:1/13:1/14:0 |
| TAG 40:3 | C_43_H_76_O_6_ | 688.56419 | 3.8432898 | 12:0/12:0/16:3 |
|  |  |  |  | 12:0/12:1/16:2 |
|  |  |  |  | 12:0/12:2/16:1 |
|  |  |  |  | 12:0/12:3/16:0 |
|  |  |  |  | 12:0/14:0/14:3 |
|  |  |  |  | 12:0/14:1/14:2 |
|  |  |  |  | 12:1/12:1/16:1 |
| TAG 40:4 | C_43_H_74_O_6_ | 686.54854 | 3.6461085 | 12:2/12:2/16:0 |
| TAG 41:0 | C_44_H_84_O_6_ | 708.6268 | 4.4554335 | 12:0/12:0/17:0 |
|  |  |  |  | 12:0/13:0/16:0 |
|  |  |  |  | 12:0/14:0/15:0 |
| TAG 41:1 | C_44_H_82_O_6_ | 706.61114 | 4.2175 | 12:0/12:0/17:1 |
|  |  |  |  | 12:0/13:0/16:1 |
|  |  |  |  | 12:0/14:0/15:1 |
| TAG 42:0 | C_45_H_86_O_6_ | 722.64244 | 4.5516388 | 12:0/14:0/16:0 |
| TAG 42:1 | C_45_H_84_O_6_ | 720.62679 | 4.3913702 | 12:0/12:0/18:1 |
| TAG 42:2 | C_45_H_82_O_6_ | 718.61114 | 4.2269388 | 12:0/12:0/18:2 |
| TAG 42:3 | C_45_H_80_O_6_ | 716.59549 | 4.0738102 | 12:0/12:0/18:3 |
|  |  |  |  | 12:0/12:1/18:2 |
|  |  |  |  | 12:0/12:2/18:1 |
| TAG 42:4 | C_45_H_78_O_6_ | 714.57984 | 3.8889 | 12:1/12:1/18:2 |
| TAG 43:0 | C_46_H_88_O_6_ | 736.6581 | 4.642433 | 12:0/14:0/17:0 |
|  |  |  |  | 12:0/15:0/16:0 |
|  |  |  |  | 14:0/14:0/15:0 |
| TAG 43:1 | C_46_H_86_O_6_ | 734.6424 | 4.435533 | 12:0/13:0/18:1 |
|  |  |  |  | 12:0/14:0/17:1 |
|  |  |  |  | 12:0/15:0/16:1 |
|  |  |  |  | 13:0/13:0/17:1 |
|  |  |  |  | 13:0/14:1/16:0 |
| TAG 43:2 | C_46_H_84_O_6_ | 732.62679 | 4.3181373 | 12:0/13:0/18:2 |
|  |  |  |  | 12:0/14:0/17:2 |
|  |  |  |  | 14:0/14:2/15:0 |
|  |  |  |  | 14:1/14:1/15:0 |
| TAG 44:0 | C_47_H_90_O_6_ | 750.67374 | 4.7345418 | 12:0/14:0/18:0 |
|  |  |  |  | 12:0/16:0/16:0 |
|  |  |  |  | 14:0/14:0/16:0 |
| TAG 44:1 | C_47_H_88_O_6_ | 748.65809 | 4.5808055 | 12:0/14:0/18:1 |
| TAG 44:2 | C_47_H_86_O_6_ | 746.64244 | 4.432536 | 12:0/14:0/18:2 |
| TAG 44:3 | C_47_H_84_O_6_ | 744.62679 | 4.2861934 | 12:0/12:0/20:3 |
|  |  |  |  | 12:0/14:0/18:3 |
|  |  |  |  | 12:0/14:1/18:2 |
| TAG 44:4 | C_47_H_82_O_6_ | 742.61114 | 4.1098003 | 12:0/12:0/20:4 |
|  |  |  |  | 12:0/14:2/18:2 |
|  |  |  |  | 12:1/12:1/20:2 |
|  |  |  |  | 12:3/14:0/18:1 |
|  |  |  |  | 13:1/13:1/18:2 |
|  |  |  |  | 14:1/14:1/16:2 |
| TAG 45:0 | C_48_H_92_O_6_ | 764.6894 | 4.8176497 | 12:0/16:0/17:0 |
|  |  |  |  | 14:0/15:0/16:0 |
| TAG 45:1 | C_48_H_90_O_6_ | 762.6737 | 4.672222 | 12:0/14:0/19:1 |
|  |  |  |  | 12:0/15:0/18:1 |
|  |  |  |  | 12:0/16:0/17:1 |
|  |  |  |  | 13:0/14:0/18:1 |
|  |  |  |  | 14:0/14:0/17:1 |
| TAG 45:2 | C_48_H_88_O_6_ | 760.6581 | 4.5296432 | 12:0/15:0/18:2 |
|  |  |  |  | 12:0/16:1/17:1 |
|  |  |  |  | 13:0/13:0/19:2 |
|  |  |  |  | 13:0/14:0/18:2 |
| TAG 46:0 | C_49_H_94_O_6_ | 778.70504 | 4.902311 | 12:0/16:0/18:0 |
|  |  |  |  | 14:0/16:0/16:0 |
| TAG 46:1 | C_49_H_92_O_6_ | 776.68939 | 4.7604555 | 12:0/16:0/18:1 |
| TAG 46:2 | C_49_H_90_O_6_ | 774.67374 | 4.620439 | 12:0/16:0/18:2 |
| TAG 46:3 | C_49_H_88_O_6_ | 772.65809 | 4.4759473 | 12:0/16:0/18:3 |
|  |  |  |  | 12:0/16:1/18:2 |
| TAG 46:4 | C_49_H_86_O_6_ | 770.64244 | 4.3186555 | 12:0/16:1/18:3 |
|  |  |  |  | 12:0/16:2/18:2 |
|  |  |  |  | 12:1/16:1/18:2 |
|  |  |  |  | 12:3/16:0/18:1 |
|  |  |  |  | 14:1/14:1/18:2 |
| TAG 46:5 | C_49_H_84_O_6_ | 768.62679 | 4.114342 | 12:0/16:2/18:3 |
|  |  |  |  | 12:3/16:0/18:2 |
|  |  |  |  | 14:0/14:2/18:3 |
|  |  |  |  | 14:0/14:3/18:2 |
|  |  |  |  | 14:1/14:1/18:3 |
| TAG 46:6 | C_49_H_82_O_6_ | 766.61114 | 4.027333 | 14:2/14:2/18:2 |
| TAG 47:0 | C_50_H_96_O_6_ | 792.7207 | 4.939933 | 14:0/16:0/17:0 |
| TAG 47:1 | C_50_H_94_O_6_ | 790.705 | 4.8393972 | 12:0/17:0/18:1 |
|  |  |  |  | 13:0/16:0/18:1 |
|  |  |  |  | 14:0/15:0/18:1 |
|  |  |  |  | 14:0/16:1/17:0 |
|  |  |  |  | 15:0/16:0/16:1 |
| TAG 47:2 | C_50_H_92_O_6_ | 788.6894 | 4.7100943 | 12:0/17:0/18:2 |
|  |  |  |  | 12:0/17:1/18:1 |
|  |  |  |  | 13:0/16:1/18:1 |
|  |  |  |  | 14:0/15:0/18:2 |
|  |  |  |  | 14:0/16:0/17:2 |
|  |  |  |  | 14:0/16:1/17:1 |
|  |  |  |  | 14:1/15:0/18:1 |
| TAG 47:3 | C_50_H_90_O_6_ | 786.6737 | 4.6387667 | 12:0/17:1/18:2 |
|  |  |  |  | 14:0/14:0/19:3 |
| TAG 48:0 | C_51_H_98_O_6_ | 806.73634 | 5.0540766 | 14:0/16:0/18:0 |
| TAG 48:1 | C_51_H_96_O_6_ | 804.72069 | 4.922639 | 14:0/16:0/18:1 |
| TAG 48:2 | C_51_H_94_O_6_ | 802.70504 | 4.7964277 | 12:0/18:1/18:1 |
|  |  |  |  | 14:0/16:0/18:2 |
|  |  |  |  | 14:0/16:1/18:1 |
| TAG 48:3 | C_51_H_92_O_6_ | 800.68939 | 4.6531335 | 12:0/18:1/18:2 |
| TAG 48:4 | C_51_H_90_O_6_ | 798.67374 | 4.511836 | 12:0/18:1/18:3 |
|  |  |  |  | 12:0/18:2/18:2 |
| TAG 48:5 | C_51_H_88_O_6_ | 796.65809 | 4.3696918 | 12:0/18:2/18:3 |
| TAG 48:6 | C_51_H_86_O_6_ | 794.64244 | 4.255775 | 12:0/14:0/22:6 |
|  |  |  |  | 12:0/18:3/18:3 |
|  |  |  |  | 12:1/18:2/18:3 |
|  |  |  |  | 12:2/18:2/18:2 |
|  |  |  |  | 14:1/14:1/20:4 |
|  |  |  |  | 15:2/15:2/18:2 |
| TAG 49:0 | C_52_H_100_O_6_ | 820.752 | 5.0925085 | 15:0/16:0/18:0 |
|  |  |  |  | 16:0/16:0/17:0 |
| TAG 49:1 | C_52_H_98_O_6_ | 818.7363 | 5.0002375 | 15:0/16:0/18:1 |
| TAG 49:2 | C_52_H_96_O_6_ | 816.7207 | 4.8755802 | 14:0/17:0/18:2 |
|  |  |  |  | 14:0/17:1/18:1 |
|  |  |  |  | 15:0/16:0/18:2 |
|  |  |  |  | 15:0/16:1/18:1 |
|  |  |  |  | 15:1/16:0/18:1 |
|  |  |  |  | 16:0/16:1/17:1 |
|  |  |  |  | 16:1/16:1/17:0 |
| TAG 49:3 | C_52_H_94_O_6_ | 814.705 | 4.7461443 | 12:0/18:2/19:1 |
|  |  |  |  | 13:0/18:1/18:2 |
|  |  |  |  | 14:0/16:0/19:3 |
|  |  |  |  | 14:0/17:1/18:2 |
|  |  |  |  | 15:0/16:0/18:3 |
|  |  |  |  | 15:0/16:1/18:2 |
|  |  |  |  | 15:1/15:1/19:1 |
|  |  |  |  | 15:1/16:1/18:1 |
| TAG 49:4 | C_52_H_92_O_6_ | 812.68939 | 4.589183 | 15:1/15:1/19:2 |
| TAG 50:0 | C_53_H_102_O_6_ | 834.76764 | 5.194786 | 16:0/16:0/18:0 |
| TAG 50:1 | C_53_H_100_O_6_ | 832.75199 | 5.0714085 | 16:0/16:0/18:1 |
| TAG 50:2 | C_53_H_98_O_6_ | 830.73634 | 4.9516112 | 16:0/16:0/18:2 |
|  |  |  |  | 16:0/16:1/18:1 |
| TAG 50:3 | C_53_H_96_O_6_ | 828.72069 | 4.8238693 | 14:0/18:1/18:2 |
|  |  |  |  | 16:0/16:1/18:2 |
| TAG 50:4 | C_53_H_94_O_6_ | 826.70504 | 4.6920434 | 14:0/18:1/18:3 |
|  |  |  |  | 14:0/18:2/18:2 |
| TAG 50:5 | C_53_H_92_O_6_ | 824.68939 | 4.5669998 | 12:0/18:1/20:4 |
|  |  |  |  | 12:0/18:2/20:3 |
|  |  |  |  | 14:0/18:2/18:3 |
| TAG 50:6 | C_53_H_90_O_6_ | 822.67374 | 4.4934723 | 12:0/16:0/22:6 |
|  |  |  |  | 12:0/18:2/20:4 |
|  |  |  |  | 12:0/18:3/20:3 |
|  |  |  |  | 14:0/14:0/22:6 |
|  |  |  |  | 14:0/18:3/18:3 |
|  |  |  |  | 14:1/18:2/18:3 |
|  |  |  |  | 14:2/18:1/18:3 |
|  |  |  |  | 14:3/18:1/18:2 |
| TAG 50:7 | C_53_H_88_O_6_ | 820.65809 | 4.3551723 | 12:0/16:1/22:6 |
|  |  |  |  | 12:1/18:1/20:5 |
|  |  |  |  | 14:1/14:1/22:5 |
| TAG 51:0 | C_54_H_104_O_6_ | 848.7833 | 5.2528 | 16:0/16:0/19:0 |
|  |  |  |  | 16:0/17:0/18:0 |
|  |  |  |  | 17:0/17:0/17:0 |
| TAG 51:1 | C_53_H_102_O_6_ | 846.7676 | 5.1407598 | 16:0/17:0/18:1 |
| TAG 51:2 | C_54_H_100_O_6_ | 844.752 | 5.0279766 | 15:0/18:1/18:1 |
|  |  |  |  | 16:0/17:0/18:2 |
|  |  |  |  | 16:0/17:1/18:1 |
| TAG 51:3 | C_54_H_98_O_6_ | 842.7363 | 4.9046333 | 15:0/18:1/18:2 |
|  |  |  |  | 16:0/17:1/18:2 |
| TAG 51:4 | C_54_H_96_O_6_ | 840.7207 | 4.7829085 | 15:0/17:2/19:2 |
|  |  |  |  | 15:0/18:2/18:2 |
| TAG 51:5 | C_54_H_94_O_6_ | 838.70504 | 4.6957 | 15:0/18:2/18:3 |
|  |  |  |  | 15:1/15:2/21:2 |
| TAG 51:6 | C_54_H_92_O_6_ | 836.68939 | 4.683678 | 15:1/15:1/21:4 |
|  |  |  |  | 15:1/17:3/19:2 |
|  |  |  |  | 17:0/17:3/17:3 |
| TAG 52:0 | C_55_H_106_O_6_ | 862.79894 | 5.3210833 | 16:0/18:0/18:0 |
| TAG 52:1 | C_55_H_104_O_6_ | 860.78329 | 5.211789 | 16:0/18:0/18:1 |
| TAG 52:2 | C_55_H_102_O_6_ | 858.76764 | 5.096853 | 16:0/18:1/18:1 |
| TAG 52:3 | C_55_H_100_O_6_ | 856.75199 | 4.9837445 | 16:0/18:1/18:2 |
| TAG 52:4 | C_55_H_98_O_6_ | 854.73634 | 4.8599502 | 16:0/18:1/18:3 |
|  |  |  |  | 16:0/18:2/18:2 |
| TAG 52:5 | C_55_H_96_O_6_ | 852.72069 | 4.7363225 | 16:0/18:2/18:3 |
|  |  |  |  | 16:1/18:2/18:2 |
| TAG 52:6 | C_55_H_94_O_6_ | 850.70504 | 4.618908 | 12:0/18:0/22:6 |
|  |  |  |  | 12:0/18:2/22:4 |
|  |  |  |  | 14:0/16:0/22:6 |
|  |  |  |  | 16:0/18:3/18:3 |
|  |  |  |  | 16:1/16:1/20:4 |
|  |  |  |  | 16:1/18:2/18:3 |
|  |  |  |  | 16:2/18:1/18:3 |
|  |  |  |  | 16:3/18:1/18:2 |
| TAG 52:7 | C_55_H_92_O_6_ | 848.68939 | 4.563392 | 12:0/18:1/22:6 |
|  |  |  |  | 12:0/18:2/22:5 |
|  |  |  |  | 14:1/18:2/20:4 |
|  |  |  |  | 16:3/18:2/18:2 |
| TAG 52:8 | C_55_H_90_O_6_ | 846.67374 | 4.380442 | 12:0/18:2/22:6 |
|  |  |  |  | 12:1/18:1/22:6 |
|  |  |  |  | 15:1/15:1/22:6 |
| TAG 53:1 | C_56_H_106_O_6_ | 874.7989 | 5.2745 | 16:0/17:0/20:1 |
|  |  |  |  | 16:0/18:0/19:1 |
|  |  |  |  | 16:0/18:1/19:0 |
|  |  |  |  | 17:0/18:0/18:1 |
| TAG 53:2 | C_56_H_104_O_6_ | 872.7833 | 5.1589555 | 16:0/18:1/19:1 |
|  |  |  |  | 17:0/17:0/19:2 |
|  |  |  |  | 17:0/18:0/18:2 |
|  |  |  |  | 17:0/18:1/18:1 |
|  |  |  |  | 17:1/18:0/18:1 |
|  |  |  |  | 17:2/18:0/18:0 |
| TAG 53:3 | C_56_H_102_O_6_ | 870.7676 | 5.0541098 | 17:0/18:1/18:2 |
|  |  |  |  | 17:1/18:1/18:1 |
| TAG 53:4 | C_56_H_100_O_6_ | 868.752 | 4.9315668 | 17:0/18:2/18:2 |
|  |  |  |  | 17:1/17:2/19:1 |
|  |  |  |  | 17:1/18:1/18:2 |
|  |  |  |  | 17:2/18:1/18:1 |
| TAG 53:5 | C_56_H_98_O_6_ | 866.7363 | 5.015234 | 15:0/17:3/21:2 |
|  |  |  |  | 15:1/17:2/21:2 |
|  |  |  |  | 17:0/18:2/18:3 |
|  |  |  |  | 17:1/18:2/18:2 |
|  |  |  |  | 17:3/18:1/18:1 |
| TAG 53:6 | C_56_H_96_O_6_ | 864.72069 | 4.8371 | 15:0/17:2/21:4 |
|  |  |  |  | 17:2/17:2/19:2 |
| TAG 53:9 | C_56_H_90_O_6_ | 858.67374 | 4.494133 | 17:3/17:3/19:3 |
| TAG 54:0 | C_57_H_110_O_6_ | 890.83024 | 5.4409753 | 16:0/18:0/20:0 |
| TAG 54:1 | C_57_H_108_O_6_ | 888.81459 | 5.3387222 | 16:0/18:0/20:1 |
|  |  |  |  | 16:0/18:1/20:0 |
|  |  |  |  | 18:0/18:0/18:1 |
| TAG 54:2 | C_57_H_106_O_6_ | 886.79894 | 5.230478 | 16:0/18:1/20:1 |
|  |  |  |  | 18:0/18:1/18:1 |
| TAG 54:3 | C_57_H_104_O_6_ | 884.78329 | 5.1233368 | 18:0/18:1/18:2 |
|  |  |  |  | 18:1/18:1/18:1 |
| TAG 54:4 | C_57_H_102_O_6_ | 882.76764 | 5.0087167 | 18:1/18:1/18:2 |
| TAG 54:5 | C_57_H_100_O_6_ | 880.75199 | 4.887036 | 18:1/18:2/18:2 |
| TAG 54:6 | C_57_H_98_O_6_ | 878.73634 | 4.7621777 | 18:1/18:2/18:3 |
|  |  |  |  | 18:2/18:2/18:2 |
| TAG 54:7 | C_57_H_96_O_6_ | 876.72069 | 4.63505 | 14:0/18:1/22:6 |
|  |  |  |  | 16:0/18:2/20:5 |
|  |  |  |  | 18:1/18:3/18:3 |
|  |  |  |  | 18:2/18:2/18:3 |
| TAG 54:8 | C_57_H_94_O_6_ | 874.70504 | 4.5091623 | 18:1/18:3/18:4 |
|  |  |  |  | 18:2/18:3/18:3 |
| TAG 55:1 | C_58_H_110_O_6_ | 902.83024 | 5.3781833 | 16:0/17:0/22:1 |
|  |  |  |  | 16:0/18:1/21:0 |
| TAG 55:2 | C_58_H_108_O_6_ | 900.81459 | 5.2521663 | 15:0/18:1/22:1 |
|  |  |  |  | 16:0/18:1/21:1 |
|  |  |  |  | 17:0/18:1/20:1 |
|  |  |  |  | 18:0/18:1/19:1 |
|  |  |  |  | 18:0/18:2/19:0 |
|  |  |  |  | 18:1/18:1/19:0 |
| TAG 55:3 | C_58_H_106_O_6_ | 898.7989 | 5.1853745 | 17:0/18:2/20:1 |
|  |  |  |  | 18:1/18:1/19:1 |
|  |  |  |  | 18:1/18:2/19:0 |
| TAG 55:5 | C_58_H_102_O_6_ | 894.7676 | 5.1175668 | 17:0/17:2/21:3 |
|  |  |  |  | 17:0/18:2/20:3 |
|  |  |  |  | 17:0/19:0/19:5 |
|  |  |  |  | 17:0/19:1/19:4 |
|  |  |  |  | 17:0/19:2/19:3 |
|  |  |  |  | 17:1/18:2/20:2 |
|  |  |  |  | 17:1/19:0/19:4 |
|  |  |  |  | 17:1/19:1/19:3 |
|  |  |  |  | 17:1/19:2/19:2 |
|  |  |  |  | 17:2/17:2/21:1 |
|  |  |  |  | 17:2/18:0/20:3 |
|  |  |  |  | 17:2/18:1/20:2 |
|  |  |  |  | 17:2/18:3/20:0 |
|  |  |  |  | 17:2/19:0/19:3 |
|  |  |  |  | 17:2/19:1/19:2 |
|  |  |  |  | 18:0/18:0/19:5 |
|  |  |  |  | 18:0/18:2/19:3 |
|  |  |  |  | 18:1/18:1/19:3 |
| TAG 55:6 | C_58_H_100_O_6_ | 892.752 | 4.959558 | 15:0/20:3/20:3 |
|  |  |  |  | 17:0/17:2/21:4 |
|  |  |  |  | 17:0/19:2/19:4 |
|  |  |  |  | 17:0/19:3/19:3 |
|  |  |  |  | 17:1/19:1/19:4 |
|  |  |  |  | 17:1/19:2/19:3 |
|  |  |  |  | 17:2/17:3/21:1 |
|  |  |  |  | 17:2/18:1/20:3 |
|  |  |  |  | 17:2/19:0/19:4 |
|  |  |  |  | 17:2/19:1/19:3 |
|  |  |  |  | 17:2/19:2/19:2 |
|  |  |  |  | 18:1/18:1/19:4 |
|  |  |  |  | 18:3/18:3/19:0 |
| TAG 55:7 | C_58_H_98_O_6_ | 890.73634 | 5.07485 | 17:1/19:3/19:3 |
| TAG 56:0 | C_59_H_114_O_6_ | 918.86154 | 5.5343835 | 16:0/18:0/22:0 |
| TAG 56:1 | C_59_H_112_O_6_ | 916.84589 | 5.4573278 | 16:0/18:0/22:1 |
|  |  |  |  | 16:0/18:1/22:0 |
|  |  |  |  | 18:0/18:1/20:0 |
| TAG 56:2 | C_59_H_110_O_6_ | 914.83024 | 5.3502534 | 16:0/18:1/22:1 |
|  |  |  |  | 16:0/18:2/22:0 |
|  |  |  |  | 18:0/18:1/20:1 |
|  |  |  |  | 18:0/18:2/20:0 |
|  |  |  |  | 18:1/18:1/20:0 |
| TAG 56:3 | C_59_H_108_O_6_ | 912.81459 | 5.249937 | 16:0/18:1/22:2 |
|  |  |  |  | 16:0/18:2/22:1 |
|  |  |  |  | 18:0/18:1/20:2 |
|  |  |  |  | 18:0/18:2/20:1 |
|  |  |  |  | 18:1/18:1/20:1 |
|  |  |  |  | 18:1/18:2/20:0 |
| TAG 56:4 | C_59_H_106_O_6_ | 910.79894 | 5.1473298 | 18:1/18:1/20:2 |
|  |  |  |  | 18:1/18:2/20:1 |
| TAG 56:5 | C_59_H_104_O_6_ | 908.78329 | 5.0407722 | 16:0/18:1/22:4 |
|  |  |  |  | 18:1/18:1/20:3 |
|  |  |  |  | 18:1/18:2/20:2 |
|  |  |  |  | 18:2/18:2/20:1 |
| TAG 56:6 | C_59_H_102_O_6_ | 906.76764 | 4.93541 | 18:0/18:2/20:4 |
|  |  |  |  | 18:1/18:1/20:4 |
|  |  |  |  | 18:1/18:2/20:3 |
|  |  |  |  | 18:1/18:3/20:2 |
| TAG 56:7 | C_59_H_100_O_6_ | 904.75199 | 4.8498504 | 16:0/18:1/22:6 |
|  |  |  |  | 18:1/18:2/20:4 |
|  |  |  |  | 18:2/18:2/20:3 |
| TAG 56:8 | C_59_H_98_O_6_ | 902.73634 | 4.7246443 | 16:0/18:2/22:6 |
|  |  |  |  | 18:2/18:2/20:4 |
|  |  |  |  | 18:2/18:3/20:3 |
| TAG 56:9 | C_59_H_96_O_6_ | 900.72069 | 4.6123835 | 16:1/18:2/22:6 |
|  |  |  |  | 18:2/18:3/20:4 |
| TAG 57:1 | C_60_H_114_O_6_ | 930.86154 | 5.503225 | 17:0/18:1/22:0 |
|  |  |  |  | 18:0/18:1/21:0 |
| TAG 57:11 | C_60_H_94_O_6_ | 910.70504 | 4.7792 | 17:3/20:4/20:4 |
| TAG 57:2 | C_60_H_112_O_6_ | 928.84589 | 5.3862415 | 15:0/21:1/21:1 |
|  |  |  |  | 16:0/19:1/22:1 |
| TAG 57:3 | C_60_H_110_O_6_ | 926.83024 | 5.3140415 | 17:0/18:2/22:1 |
|  |  |  |  | 18:1/18:2/21:0 |
| TAG 57:6 | C_60_H_108_O_6_ | 924.81459 | 5.115684 | 18:1/18:1/21:4 |
| TAG 58:0 | C_61_H_118_O_6_ | 946.89284 | 5.658333 | 18:0/20:0/20:0 |
| TAG 58:1 | C_61_H_116_O_6_ | 944.87719 | 5.5681832 | 16:0/20:0/22:1 |
|  |  |  |  | 16:0/20:1/22:0 |
|  |  |  |  | 18:0/18:0/22:1 |
|  |  |  |  | 18:0/18:1/22:0 |
|  |  |  |  | 18:1/20:0/20:0 |
| TAG 58:10 | C_61_H_98_O_6_ | 926.73634 | 4.6802 | 18:2/18:2/22:6 |
| TAG 58:2 | C_61_H_114_O_6_ | 942.86154 | 5.4760585 | 16:0/20:1/22:1 |
|  |  |  |  | 18:0/18:1/22:1 |
|  |  |  |  | 18:0/18:2/22:0 |
|  |  |  |  | 18:1/18:1/22:0 |
| TAG 58:3 | C_61_H_112_O_6_ | 940.84589 | 5.37183 | 18:0/18:2/22:1 |
|  |  |  |  | 18:1/18:1/22:1 |
|  |  |  |  | 18:1/18:2/22:0 |
| TAG 58:4 | C_61_H_110_O_6_ | 938.83024 | 5.26847 | 18:1/18:1/22:2 |
|  |  |  |  | 18:1/18:2/22:1 |
| TAG 58:5 | C_61_H_108_O_6_ | 936.81459 | 5.1736934 | 18:0/18:1/22:4 |
|  |  |  |  | 18:1/18:2/22:2 |
|  |  |  |  | 18:1/18:3/22:1 |
|  |  |  |  | 18:1/20:2/20:2 |
|  |  |  |  | 18:2/18:2/22:1 |
|  |  |  |  | 18:2/20:1/20:2 |
| TAG 58:6 | C_61_H_106_O_6_ | 934.79894 | 5.0815277 | 18:0/18:2/22:4 |
|  |  |  |  | 18:1/18:1/22:4 |
|  |  |  |  | 18:1/18:2/22:3 |
|  |  |  |  | 18:2/18:3/22:1 |
| TAG 58:7 | C_61_H_104_O_6_ | 932.78329 | 4.9747223 | 18:0/18:1/22:6 |
|  |  |  |  | 18:1/18:1/22:5 |
|  |  |  |  | 18:1/18:2/22:4 |
| TAG 58:8 | C_61_H_102_O_6_ | 930.76764 | 4.933711 | 18:0/18:2/22:6 |
|  |  |  |  | 18:1/18:1/22:6 |
|  |  |  |  | 18:1/18:2/22:5 |
| TAG 58:9 | C_61_H_100_O_6_ | 928.75199 | 4.8081415 | 18:1/18:2/22:6 |
|  |  |  |  | 18:2/18:2/22:5 |
| TAG 59:3 | C_62_H_114_O_6_ | 954.86154 | 5.435233 | 18:2/19:0/22:1 |
| TAG 60:1 | C_63_H_120_O_6_ | 972.90849 | 5.6698167 | 16:0/22:0/22:1 |
|  |  |  |  | 18:0/20:0/22:1 |
|  |  |  |  | 18:1/20:0/22:0 |
|  |  |  |  | 18:1/21:0/21:0 |
| TAG 60:10 | C_63_H_102_O_6_ | 954.76764 | 4.7582 | 18:2/20:3/22:5 |
| TAG 60:2 | C_63_H_118_O_6_ | 970.89284 | 5.5783666 | 16:0/22:1/22:1 |
|  |  |  |  | 18:0/20:2/22:0 |
|  |  |  |  | 18:1/20:0/22:1 |
|  |  |  |  | 18:1/20:1/22:0 |
| TAG 60:3 | C_63_H_116_O_6_ | 968.87719 | 5.48342 | 18:0/20:1/22:2 |
|  |  |  |  | 18:0/20:3/22:0 |
|  |  |  |  | 18:1/20:0/22:2 |
|  |  |  |  | 18:1/20:1/22:1 |
|  |  |  |  | 18:1/20:2/22:0 |
|  |  |  |  | 18:1/21:1/21:1 |
|  |  |  |  | 18:2/20:0/22:1 |
|  |  |  |  | 18:2/20:1/22:0 |
|  |  |  |  | 20:1/20:1/20:1 |
| TAG 60:4 | C_63_H_114_O_6_ | 966.86154 | 5.391975 | 16:0/22:2/22:2 |
|  |  |  |  | 18:1/20:1/22:2 |
|  |  |  |  | 18:1/20:2/22:1 |
|  |  |  |  | 18:1/20:3/22:0 |
|  |  |  |  | 18:2/20:1/22:1 |
|  |  |  |  | 18:2/20:2/22:0 |
|  |  |  |  | 18:2/21:1/21:1 |
|  |  |  |  | 20:1/20:1/20:2 |
| TAG 60:5 | C_63_H_112_O_6_ | 964.84589 | 5.2988613 | 18:1/20:1/22:3 |
|  |  |  |  | 18:1/20:2/22:2 |
|  |  |  |  | 18:1/20:3/22:1 |
|  |  |  |  | 18:2/20:2/22:1 |
|  |  |  |  | 18:3/20:2/22:0 |
|  |  |  |  | 20:1/20:1/20:3 |
|  |  |  |  | 20:1/20:2/20:2 |
| TAG 60:6 | C_63_H_110_O_6_ | 962.83024 | 5.2126723 | 18:1/20:0/22:5 |
|  |  |  |  | 18:1/20:1/22:4 |
|  |  |  |  | 18:1/20:3/22:2 |
|  |  |  |  | 18:1/20:4/22:1 |
|  |  |  |  | 18:2/20:2/22:2 |
|  |  |  |  | 18:2/20:3/22:1 |
| TAG 60:7 | C_63_H_108_O_6_ | 960.81459 | 5.1030496 | 18:1/20:1/22:5 |
|  |  |  |  | 18:1/20:3/22:3 |
|  |  |  |  | 18:2/20:4/22:1 |
| TAG 60:8 | C_63_H_106_O_6_ | 958.79894 | 5.016133 | 18:1/20:3/22:4 |
| TAG 62:2 | C_65_H_122_O_6_ | 998.92414 | 5.6734 | 18:0/22:1/22:1 |
|  |  |  |  | 20:1/20:1/22:0 |
| TAG 62:3 | C_65_H_120_O_6_ | 996.90849 | 5.5849168 | 18:1/22:1/22:1 |
| TAG 62:4 | C_65_H_118_O_6_ | 994.89284 | 5.5035755 | 18:2/22:1/22:1 |
|  |  |  |  | 20:1/20:1/22:2 |
| TAG 62:5 | C_65_H_116_O_6_ | 992.87719 | 5.3881833 | 18:1/22:2/22:2 |
|  |  |  |  | 18:3/22:1/22:1 |
|  |  |  |  | 20:2/20:2/22:1 |
| TAG 62:6 | C_65_H_114_O_6_ | 990.86154 | 5.341417 | 18:1/22:1/22:4 |
| TAG 62:7 | C_65_H_112_O_6_ | 988.84589 | 5.23695 | 18:0/22:1/22:6 |
|  |  |  |  | 18:1/22:2/22:4 |
|  |  |  |  | 18:2/22:1/22:4 |
| TAG 62:8 | C_65_H_110_O_6_ | 986.83024 | 5.206917 | 18:1/22:1/22:6 |
| TAG 64:2 | C_67_H_126_O_6_ | 1026.95544 | 5.764875 | 20:0/22:1/22:1 |
| TAG 64:3 | C_67_H_124_O_6_ | 1024.93979 | 5.68355 | 20:0/22:1/22:2 |
|  |  |  |  | 20:1/22:1/22:1 |
| TAG 64:4 | C_67_H_122_O_6_ | 1022.92414 | 5.6108165 | 20:2/22:0/22:2 |
|  |  |  |  | 20:2/22:1/22:1 |
| TAG 64:5 | C_67_H_120_O_6_ | 1020.90849 | 5.5073665 | 20:1/22:0/22:4 |
|  |  |  |  | 20:2/22:1/22:2 |
| TAG 66:3 | C_69_H_128_O_6_ | 1052.97109 | 5.788567 | 22:1/22:1/22:1 |
| TAG 66:5 | C_69_H_124_O_6_ | 1048.93979 | 5.63375 | 22:1/22:2/22:2 |
| TAG 66:8 | C_69_H_118_O_6_ | 1042.89284 | 5.385767 | 22:2/22:2/22:4 |
| FFA 10:0 | C_10_H_20_O_2_ | 172.1463 | 0.79 | 10:0 |
| FFA 12:0 | C_12_H_24_O_2_ | 200.1776 | 0.87 | 12:0 |
| FFA 14:0 | C_14_H_28_O_2_ | 228.2089 | 1.00 | 14:0 |
| FFA 14:1 | C_14_H_26_O_2_ | 226.1933 | 0.69 | 14:1 |
| FFA 16:0 | C_16_H_32_O_2_ | 256.2402 | 1.18 | 16:0 |
| FFA 16:1 | C_16_H_30_O_2_ | 254.2246 | 1.03 | 16:1 |
| FFA 18:0 | C_18_H_36_O_2_ | 284.2715 | 1.43 | 18:0 |
| FFA 18:1 | C_18_H_34_O_2_ | 282.2559 | 1.23 | 18:1 |
| FFA 18:2 | C_18_H_32_O_2_ | 280.2402 | 1.08 | 18:2 |
| FFA 18:3 | C_18_H_30_O_2_ | 278.2246 | 0.98 | 18:3 |
| FFA 20:0 | C_20_H_40_O_2_ | 312.3028 | 1.77 | 20:0 |
| FFA 20:1 | C_20_H_38_O_2_ | 310.2872 | 1.49 | 20:1 |
| FFA 20:2 | C_20_H_36_O_2_ | 308.2715 | 1.29 | 20:2 |
| FFA 20:3 | C_20_H_34_O_2_ | 306.2559 | 1.15 | 20:3 |
| FFA 20:4 | C_20_H_32_O_2_ | 304.2402 | 1.05 | 20:4 |
| FFA 20:5 | C_20_H_30_O_2_ | 302.2246 | 0.94 | 20:5 |
| FFA 22:0 | C_22_H_44_O_2_ | 340.3341 | 2.12 | 22:0 |
| FFA 22:1 | C_22_H_42_O_2_ | 338.3185 | 1.81 | 22:1 |
| FFA 22:2 | C_22_H_40_O_2_ | 336.3028 | 1.55 | 22:2 |
| FFA 22:4 | C_22_H_36_O_2_ | 332.2715 | 1.22 | 22:4 |
| FFA 22:6 | C_22_H_32_O_2_ | 328.2402 | 0.98 | 22:6 |
| FFA 24:0 | C_24_H_48_O_2_ | 368.3654 | 2.52 | 24:0 |
| FFA 24:1 | C_24_H_46_O_2_ | 366.3498 | 2.16 | 24:1 |

The symbol “/” denotes only the fatty acid composition of lipid species, and not their sn-1, sn-2 and sn-3 ositions on the glyceride-backbone.

**Table S3 List of identified gangliosides in human milk detected by UPLC-Q-TOF-MS**

| Lipid | Formula | m/z | Retention time | value |
| --- | --- | --- | --- | --- |
| GM3 32:1;2 | C_55_H_100_N_2_O_21_ | 1124.6819 | 3.07 | d32:1 |
| GM3 34:0;2 | C_57_H_106_N_2_O_21_ | 1154.7288 | 3.06 | d34:0 |
| GM3 34:0;3 | C_57_H_106_N_2_O_22_ | 1170.7237 | 2.87 | t34:0 |
| GM3 34:1;2 | C_57_H_104_N_2_O_21_ | 1152.7132 | 3.25 | d34:1 |
| GM3 34:1;3 | C_57_H_104_N_2_O_22_ | 1168.7081 | 3.21 | t34:1 |
| GM3 34:2;3 | C_57_H_102_N_2_O_22_ | 1166.6924 | 2.41 | t34:2 |
| GM3 36:0;3 | C_59_H_110_N_2_O_22_ | 1198.7550 | 4.07 | t36:0 |
| GM3 36:1;2 | C_59_H_108_N_2_O_21_ | 1180.7445 | 4.01 | d36:1 |
| GM3 36:1;3 | C_59_H_108_N_2_O_22_ | 1196.7394 | 3.42 | t36:1 |
| GM3 36:2;2 | C_59_H_106_N_2_O_21_ | 1178.7288 | 3.54 | d36:2 |
| GM3 38:0;2 | C_61_H_114_N_2_O_21_ | 1210.7914 | 4.47 | d38:0 |
| GM3 38:0;3 | C_61_H_114_N_2_O_22_ | 1226.7863 | 4.05 | t38:0 |
| GM3 38:1;2 | C_61_H_112_N_2_O_21_ | 1208.7758 | 4.68 | d38:1 |
| GM3 38:2;2 | C_61_H_110_N_2_O_21_ | 1206.7601 | 4.20 | d38:2 |
| GM3 40:0;2 | C_63_H_118_N_2_O_21_ | 1238.8227 | 5.23 | d40:0 |
| GM3 40:0;3 | C_63_H_118_N_2_O_22_ | 1254.8176 | 5.09 | t40:0 |
| GM3 40:1;2 | C_63_H_116_N_2_O_21_ | 1236.8071 | 5.72 | d40:1 |
| GM3 40:1;3 | C_63_H_116_N_2_O_22_ | 1252.8020 | 4.08 | t40:1 |
| GM3 40:2;2 | C_63_H_114_N_2_O_21_ | 1234.7914 | 5.07 | d40:2 |
| GM3 40:2;3 | C_63_H_114_N_2_O_22_ | 1250.7863 | 3.44 | t40:2 |
| GM3 42:0;3 | C_65_H_122_N_2_O_22_ | 1282.8489 | 6.11 | t42:0 |
| GM3 42:1;2 | C_65_H_120_N_2_O_21_ | 1264.8384 | 6.74 | d42:1 |
| GM3 42:1;3 | C_65_H_120_N_2_O_22_ | 1280.8333 | 5.07 | t42:1 |
| GM3 42:2;2 | C_65_H_118_N_2_O_21_ | 1262.8227 | 5.81 | d42:2 |
| GM3 44:1;2 | C_67_H_124_N_2_O_21_ | 1292.8697 | 7.36 | d44:1 |
| GM3 44:2;2 | C_67_H_122_N_2_O_21_ | 1290.8540 | 6.94 | d44:2 |
| GD3 34:1;2 | C_68_H_121_N_3_O_29_ | 1443.8086 | 3.24 | d34:1 |
| GD3 36:1;2 | C_70_H_125_N_3_O_29_ | 1471.8399 | 3.88 | d36:1 |
| GD3 38:0;2 | C_72_H_131_N_3_O_29_ | 1501.8868 | 4.61 | d38:0 |
| GD3 38:0;3 | C_72_H_131_N_3_O_30_ | 1517.8817 | 3.71 | t38:0 |
| GD3 38:1;2 | C_72_H_129_N_3_O_29_ | 1499.8712 | 4.47 | d38:1 |
| GD3 40:0;3 | C_74_H_135_N_3_O_30_ | 1545.9130 | 5.20 | t40:0 |
| GD3 40:1;2 | C_74_H_133_N_3_O_29_ | 1527.9025 | 5.17 | d40:1 |
| GD3 40:2;2 | C_74_H_131_N_3_O_29_ | 1525.8868 | 4.60 | d40:2 |
| GD3 40:2;3 | C_74_H_131_N_3_O_30_ | 1541.8817 |  | t40:2 |
| GD3 42:0;2 | C_76_H_139_N_3_O_29_ | 1557.9494 | 6.90 | d42:0 |
| GD3 42:0;3 | C_76_H_139_N_3_O_30_ | 1573.9443 | 6.59 | t42:0 |
| GD3 42:1;2 | C_76_H_137_N_3_O_29_ | 1555.9338 | 6.76 | d42:1 |
| GD3 42:2;2 | C_76_H_135_N_3_O_29_ | 1553.9181 | 5.51 | d42:2 |

Value refers to the sum of carbon atoms and the number of double bonds attached to the dihydroxysphingosine base and their fatty acid moieties.

The “d” and “t” denotes the number of hydroxyl groups link on gangliosides, “d” refers to two hydroxyl groups and the “t” refers to three hydroxyl groups.
